# Molecular Determinants and Dynamics of Hepatitis C Virus Secretion

**DOI:** 10.1371/journal.ppat.1002466

**Published:** 2012-01-05

**Authors:** Kelly E. Coller, Nicholas S. Heaton, Kristi L. Berger, Jacob D. Cooper, Jessica L. Saunders, Glenn Randall

**Affiliations:** Department of Microbiology, The University of Chicago, Chicago, Illinois, United States of America; University of Southern California, United States of America

## Abstract

The current model of hepatitis C virus (HCV) production involves the assembly of virions on or near the surface of lipid droplets, envelopment at the ER in association with components of VLDL synthesis, and egress via the secretory pathway. However, the cellular requirements for and a mechanistic understanding of HCV secretion are incomplete at best. We combined an RNA interference (RNAi) analysis of host factors for infectious HCV secretion with the development of live cell imaging of HCV core trafficking to gain a detailed understanding of HCV egress. RNAi studies identified multiple components of the secretory pathway, including ER to Golgi trafficking, lipid and protein kinases that regulate budding from the *trans*-Golgi network (TGN), VAMP1 vesicles and adaptor proteins, and the recycling endosome. Our results support a model wherein HCV is infectious upon envelopment at the ER and exits the cell via the secretory pathway. We next constructed infectious HCV with a tetracysteine (TC) tag insertion in core (TC-core) to monitor the dynamics of HCV core trafficking in association with its cellular cofactors. In order to isolate core protein movements associated with infectious HCV secretion, only trafficking events that required the essential HCV assembly factor NS2 were quantified. TC-core traffics to the cell periphery along microtubules and this movement can be inhibited by nocodazole. Sub-populations of TC-core localize to the Golgi and co-traffic with components of the recycling endosome. Silencing of the recycling endosome component Rab11a results in the accumulation of HCV core at the Golgi. The majority of dynamic core traffics in association with apolipoprotein E (ApoE) and VAMP1 vesicles. This study identifies many new host cofactors of HCV egress, while presenting dynamic studies of HCV core trafficking in infected cells.

## Author Summary

The current model of HCV egress is that virions assemble at lipid droplets, envelope at the ER and then likely exit the hepatocyte via the secretory pathway in association with apolipoproteins. To gain a more detailed insight into infectious HCV release, we combined an RNAi analysis of host factors that are required for infectious HCV secretion with live cell imaging of HCV core trafficking. Using this approach, we identified numerous components of the secretory pathway that are both required for infectious HCV release and co-traffic with HCV core. The dynamics of HCV core trafficking, both in terms of frequency of transport, particle velocity, and the corresponding run lengths were quantified. We observe that dynamic core movements in the periphery require NS2, a viral protein required for virion assembly. Core co-traffics with multiple components of the secretory pathway, including the Golgi, recycling endosome, microtubules, VAMP1 secretory vesicles, and ApoE. This study identifies new molecular determinants of HCV secretion and describes the dynamics of their movements with HCV core in real time.

## Introduction

Hepatitis C virus (HCV) is a member of the *Flaviviridae* family that contains a single-stranded positive RNA genome (∼9600bp). This is translated into a polyprotein, which is processed by viral and host proteases into 3 structural proteins (core and the glycoproteins, E1 and E2) and 7 non-structural (NS) proteins (p7, NS2, NS3, NS4A, NS4B, NS5A, NS5B). The discovery of HCV strains that are infectious in cell culture has enhanced the appreciation of the functions of these proteins in all stages of the viral life cycle [1]–[4]. Infection of hepatocytes in cell culture requires at least four potential receptor molecules, CD81, SR-B1, claudins, and occludin [5]–[8]. Additionally, numerous entry cofactors involved in receptor-mediated clathrin endocytosis are required [9]–[11]. Following endosomal acidification, genomic RNA is released into the cytoplasm where initial translation and replication occur. Replication is associated membranous structures derived from the endoplasmic reticulum (ER) called the membranous web [12], [13].

Following replication, genomic RNAs in complex with viral and possibly cellular proteins are thought to transit to lipid droplets (LD), where core protein localizes and virion assembly occurs [14]–[18]. Although primary functions of the NS proteins include viral RNA replication and host cell interactions, several NS proteins, including p7, NS2, NS3, and NS5A are also implicated in HCV assembly [19]–[27]. NS2 is a central regulator in capsid assembly that appears to act as a scaffold to coordinate interactions between the structural and non-structural proteins leading to viral RNA encapsidation [19], [22], [23]. Capsid envelopment is thought to occur at the ER, in close juxtaposition to LDs, based on the subcellular localization of the E1 and E2 glycoproteins [28], [29].

The events following HCV assembly are less clear. The egress of flaviviruses through the secretory pathway is well documented and may be similar to the mechanism by which HCV is secreted [30], [31]. However, HCV is distinct from the flaviviruses in that HCV particles are infectious upon envelopment and appear to be secreted from the cell in association with distinct lipid and apolipoprotein associations [32]–[34]. HCV particles circulating in the blood of infected patients are rich in triglycerides, apolipoprotein B (ApoB), and apolipoprotein E (ApoE) [32], [35]–[38]. The low-density lipoprotein (LDL)-virus complex [39] possesses a broad buoyant density, ranging between 1.03-1.25g/mL. Cell culture-derived virus also has a broad range of buoyant densities, with the lower density fractions containing virus highly infectious virus [34], [35], [40]. Purified HCV virions have diverse morphologies ranging from 30-125 nm and a lipid composition resembling very low-density lipoprotein (VLDL) [34], [40]. The VLDL synthesis pathway has been implicated in infectious HCV secretion. Small interfering RNAs (siRNAs) or inhibitors that target components of VLDL synthesis, including microsomal triglyceride transfer protein, ApoB, and ApoE inhibit infectious HCV secretion [32], [33], [35], [36], [41], [42].

This study is a continuation of two previous RNAi studies in which membrane trafficking pathways were interrogated for their roles in HCV entry and replication [43], [44]. In this study, we identified numerous host cofactors that are required for infectious HCV secretion, but not HCV entry or replication. We next developed a live cell imaging approach to study HCV core trafficking using an infectious HCV that contains a tetracysteine (TC) tag insertion in the core protein (TC-core). This approach has been successfully used before to visualize single virion HIV-1 budding [45]–[47] and vesicular stomatitis virus (VSV) endocytosis [48], [49] without major defects in virus infectivity. TC-core movements that are dependent upon successful virion assembly were microtubule-dependent and associated with several components of the secretory pathway that were identified in the RNAi analysis.

## Results

### Host genes involved in HCV secretion

This study is a part of a larger analysis, which interrogated a previously described unique siRNA library that targeted known membrane trafficking pathways [43], [44]. The library consisted of 122 siRNAs targeted to membrane trafficking genes from Dharmacon, Inc and was supplemented with 18 additional genes important for replication and membrane organization in other viral systems [44]. We first identified siRNAs that inhibited infectious extracellular HCV accumulation in a primary screen and then re-tested these siRNAs in secondary assays to identify the stage of the life cycle that they influenced. Genes involved in entry had a secondary phenotype in inhibiting HCV pseudoparticle (HCVpp) infection [43] and genes involved in HCV replication had a defect in subgenomic replicon replication [44].

The primary screen was performed by electroporating four individual siRNAs per gene into Huh-7.5 cells and then allowing silencing for 48 or 72 hours before infection with HCV. Viral supernatants were collected at two days post infection (dpi) and viral titers were quantified via limiting dilution in at least two independent experiments, measured in duplicate, and normalized to at least 4 replicates of cells silenced with an irrelevant siRNA (siIRR) (Table 1). The siRNAs that inhibited extracellular infectious HCV production by more than 1 standard deviation of siIRR treated cells (>65% inhibition) were classified as “hits” and were characterized in secondary assays. To limit the possibility of siRNA off-target effects, individual siRNAs then were tested. We quantified cell viability using a luminescence-based assay that measures cellular ATP levels, following 5 days of silencing to eliminate phenotypes resulting from significantly decreased viability (*p*<0.001) (Figure S1).

**Table 1:** siRNAs perturbing infectious HCV secretion.

	Percent Inhibition*			
Gene	Infectious HCV	Host Gene RNA	Intra-:Extra- cellular virus	Function
SAR1A	80	64	4.10	ER -> Golgi
CYTH3	87	75	3.35	Golgi function
CLINT1	77	70	2.99	Golgi sorting
PI4KB	90	76	13.73	Golgi sorting
PRKD1	73	75	3.43	Golgi sorting
AP1M1	85	81	2.32	Golgi Sorting
PACSIN3	76	91	2.71	Vesicle trafficking
ARF3	75	73	2.18	Golgi trafficking
RAB11A	82	61	3.51	Exocytosis
RAB3D	69	80	9.94	Exocytosis
VAMP1	81	77	5.04	Exocytosis
RHOA	75	71	2.65	Actin/remodeling
GIT1	71	69	3.04	Actin/remodeling
WAS	71	91	5.87	Actin/remodeling

*as compared to siIRRelevant treated cells.

In this study, genes that were required for infectious HCV production, but not HCVpp entry or replicon replication, were further tested for a role in infectious HCV secretion. We repeated the RNAi analysis and quantified intra- and extra-cellular HCV infectivity following HCV infection of siRNA-treated cells. Table 1 shows the ratio of intra- to extra-cellular virus, with a value greater than 1 indicating a defect in extracellular infectious HCV release. The titer of intracellular virus did not decrease significantly in these siRNA treatments as compared to siIRR (Figure S2), indicating the genes (Table 1) are not involved in HCV assembly, but rather the secretion of infectious particles.

The siRNA screen identified multiple components of the secretory pathway, from ER to Golgi trafficking (SAR1A), to Golgi structure and function (CYTH3), cargo sorting and vesicle budding from TGN (PRKD1, AP1M1, PI4KB, CLINT1), regulators of TGN to plasma membrane trafficking (RAB11A, RAB3D, PACSIN3), and the v-snare, VAMP1. This suggests that HCV virions egress through the secretory pathway from the TGN to recycling endosomes to the plasma membrane. A role for ER to Golgi trafficking genes, such as SAR1A, ARF3, and CYTH3, in the egress of infectious HCV is supported by previous data showing that brefeldin A treatment of infected cells accumulates intracellular infectious HCV [35]. Additional genes cluster into actin and membrane remodeling pathways (RHOA, GIT1, WAS).

### Construction and characterization of fluorescently labeled HCV core

**Figure 1:**
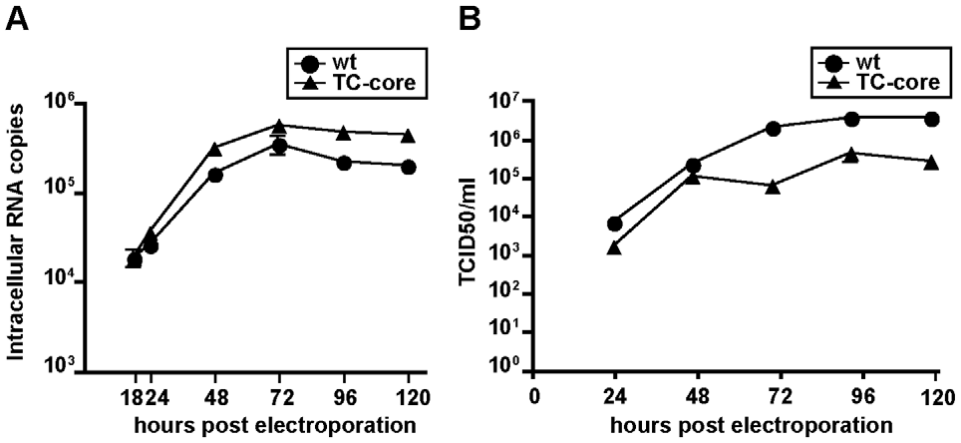
Characterization of tetracysteine (TC) tag insertion in core. A. Replication data for WT (J6/JFH1) and TC-core viruses. Time points indicated. Error bar, standard deviation. B. Titer data for WT and TC-core viruses. Viral supernatants were collected at indicated timepoints and titered by limiting dilution assay. Shown are the averages of 4 sets of titer data. Error bar, standard deviation.

To study the dynamics of HCV core trafficking in Huh-7.5 cells, a TC tag was fused in frame following amino acid three of the structural protein core in a derivative of J6/JFH-1, a chimeric genotype 2a isolate. The TC sequence of CCPGCC can coordinate biarsenical dyes, FlAsH and ReAsH. The TC-core virus replicates comparably to wild type (Figure 1A), with a mild impairment of infectious virus production (Figure 1B). ReAsH treatment of TC-core infected cells does not impair infectious virus production (Figure S3).

**Figure 2:**
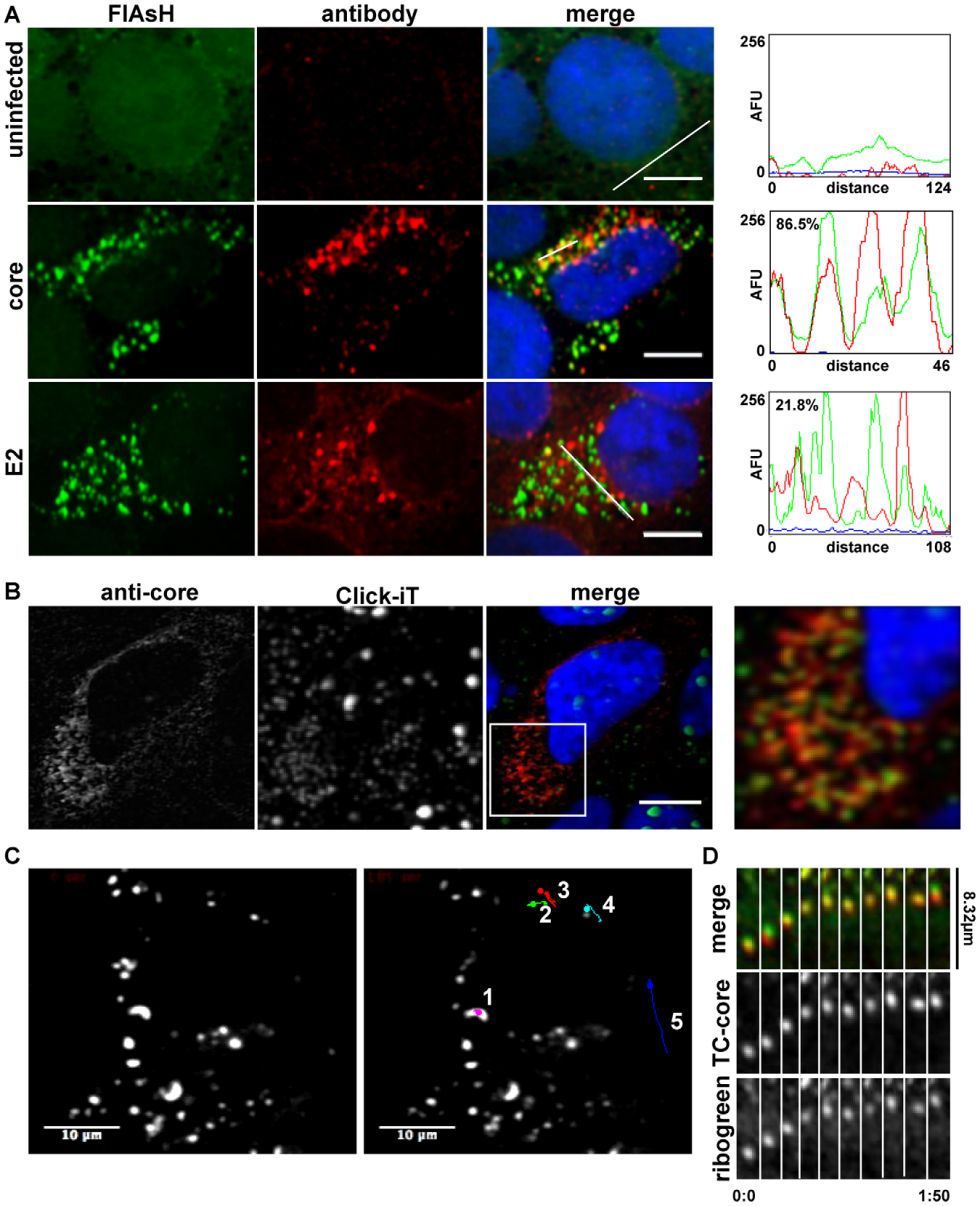
Characterization of TC-core puncta. A. Huh-7.5 cells were electroporated with TC-core RNA. At 72 hours post electroporation (hpe), cells were stained with FlAsH (1.25 µM, green) then fixed and stained for immunofluorescence using anti-core or anti-E2 antibodies (red). Scale bar is 10 microns. Fluorescence profiles of lined area in A. Distance is in pixels and intensity is in arbitrary fluorescence units (AFU). Percent colocalization of FlAsH stained puncta positive for anti-core or anti-E2 per cell is shown is upper left corner of fluorescence profile. B. Huh-7.5 cells were infected with HCV for 72 hours, treated with Click-iT in the presence of actinomycin D (1 µg/mL) to detect RNA, fixed, and probed with core antibody. C. *Left:* Single frame of an acquired fluorescent timelapse where DsRed exposures were taken every 2 seconds. *Right:* Single frame image where 5 particles were tracked and the trajectories are superimposed. Elapsed time between images is 100 seconds. Video S1. Scale bar is 10 µm. D. TC-core RNAs were electroporated into Huh-7.5 cells, maintained for 72 hours, stained with ReAsh (red) and Ribogreen (1 µl/ml), and visualized by live cell fluorescence microscopy. A representative montage of TC-core puncta transporting with RNA is shown. Elapsed time is 1 minute and 50 seconds seconds and the distance traveled is approximately 8.32 µm. Video S12.

We first tested the specificity of labeling by examining whether FlAsH TC-core fluorescence overlapped with core detected by standard immunofluorescence. TC-core RNA was electroporated into Huh-7.5 cells and three days later the cells were treated with FlAsH biarsenical dye, then processed for immunofluorescence using a monoclonal antibody against HCV core (Figure 2A). Both antibody and FlAsH dye detected the same punctate structures located in the cytoplasm of infected cells and were not present in uninfected cells. Quantitation of FlAsH and anti-core puncta determined that 86.5% of the TC-core FlAsH signal overlapped with the core antibody fluorescence (Figure 2B), demonstrating specificity of the FlAsH staining.

The localization of HCV E2 relative to TC-core was analyzed by probing for TC-core with FlAsH dye and processing for immunofluorescence with a monoclonal antibody against E2 (Figure 2A). E2 expressed from the TC-core virus had the expected reticular pattern and was also found on a subset of cytoplasmic core puncta (Figure 2A) when we used an E2 antibody that detects E2 on assembled viral particles [3], [50], 21.8% of the TC-core signal overlapped with that of E2 fluorescence (Figure 2B). Some TC-core structures that were positive for E2 were found in the cell periphery, suggesting they may represent intracellular assembled viral particles. We then investigated whether we can visualize core and RNA, as would be expected for *bona fide* HCV virions. RNA localization was examined in wild type infected cells using “Click-iT” technology (Invitrogen) and cells were stained using an anti-core antibody (Figure 2B). We observe that 20.1% percent of anti-core colocalizes with RNA. Thus, sub-populations of core are associated with E2 and RNA, although we cannot conclude whether the observed RNA represents HCV genomes.

Next, we imaged TC-core movement in living cells. Huh-7.5 cells were electroporated with TC-core RNA, maintained for 72 hours, and stained with ReAsH dye followed by imaging using confocal microscopy. We chose to image at 72 hours post electroporation because TC-core was highly expressed and detectable by biarsenical dye staining and antibody detection (Figure 2A), maximal amount of virus was released as assessed by viral titer assay (Figure 1B), and HCV induced lipid droplet relocalization to the microtubule organizing center should have occurred indicating assembly of particles was initiated [15]. Several TC-core particles are detectable in a whole cell image (Figure 2C) and a sub-population of particles displayed various movements at 72 hours post electroporation. The movements were either roughly static (Figure 2C particle 1), saltatory with many reversals in direction (Figure 2C particles 2–4), or directional movement (Figure 2C particle 5, Video S1). We then stained TC-core expressing cells with a RNA selective dye, Ribogreen, processed for ReAsH visualization of TC-core and then imaged in live cells. We observe co-trafficking of TC-core with the RNA dye, indicating that dynamic TC-core puncta associate with RNA (Figure 2D, Video S12).

### NS2 is required for dynamic TC-core movements

**Figure 3:**
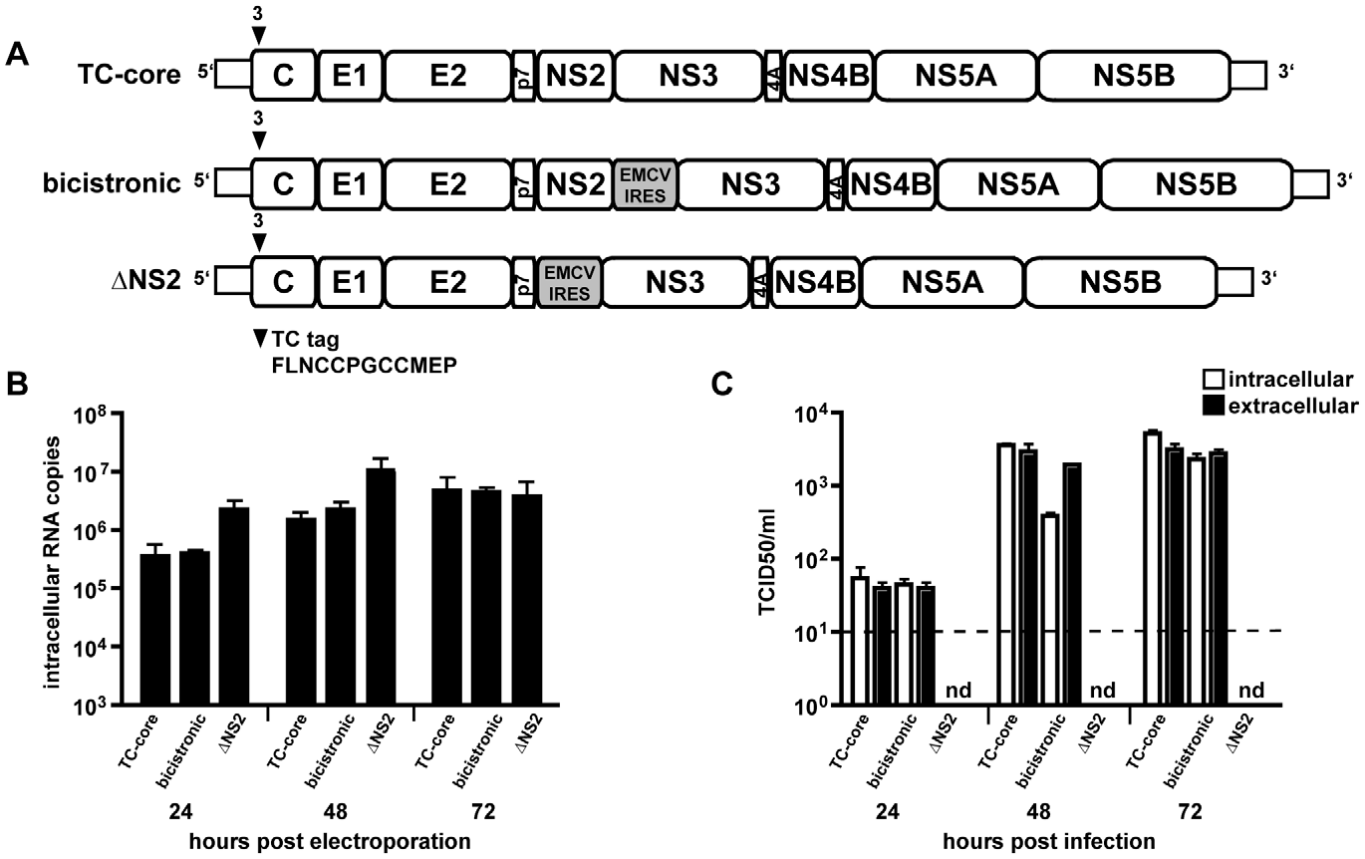
Construction and characterization of assembly mutants in TC-core background. A. Drawing indicating insertion site of TC-tag into core after amino acid 3. Shown are the TC-core, bicistronic, and NS2 deletion (ΔNS2) viruses. B. Replication data for TC-core, bicistronic, and ΔNS2 viruses. Time points indicated. Error bar, standard deviation. C. Intra- and extra-cellular infectious virus production for TC-core, bicistronic, and ΔNS2 RNAs. Virus was collected at indicated timepoints and titered by limiting dilution assay. Dotted line indicates detection limit for titer assay. nd, not detectable. Error bar, standard deviation.

We next tested whether the sub-population of dynamic TC-core movements require productive HCV virion assembly. As discussed earlier, multiple groups have published that HCV nonstructural (NS) protein 2, NS2, is required for HCV assembly by acting as a scaffold bridging structural and non-structural proteins [19], [22], [23], [51]. The function of NS2 in assembly does not require its juxtaposition next to NS3 in the viral genome, such that HCV RNAs with an encephalomyocarditis virus (EMCV) internal ribosome entry site (IRES) inserted between NS2 and NS3 are infectious [52]. Therefore, we constructed two additional viruses, a bicistronic HCV with the TC-core tag and a bicistronic TC-core version with a deleted NS2 (ΔNS2) (Figure 3A). RNAs were transcribed *in vitro* and electroporated into Huh-7.5 cells, then RNA replication and intra- and extra-cellular infectious virus were quantified over a time course. As expected, both bicistronic constructs were replication competent, (Figure 3B), while only the bicistronic virus containing NS2 was capable of producing infectious virus (Figure 3C).

**Figure 4:**
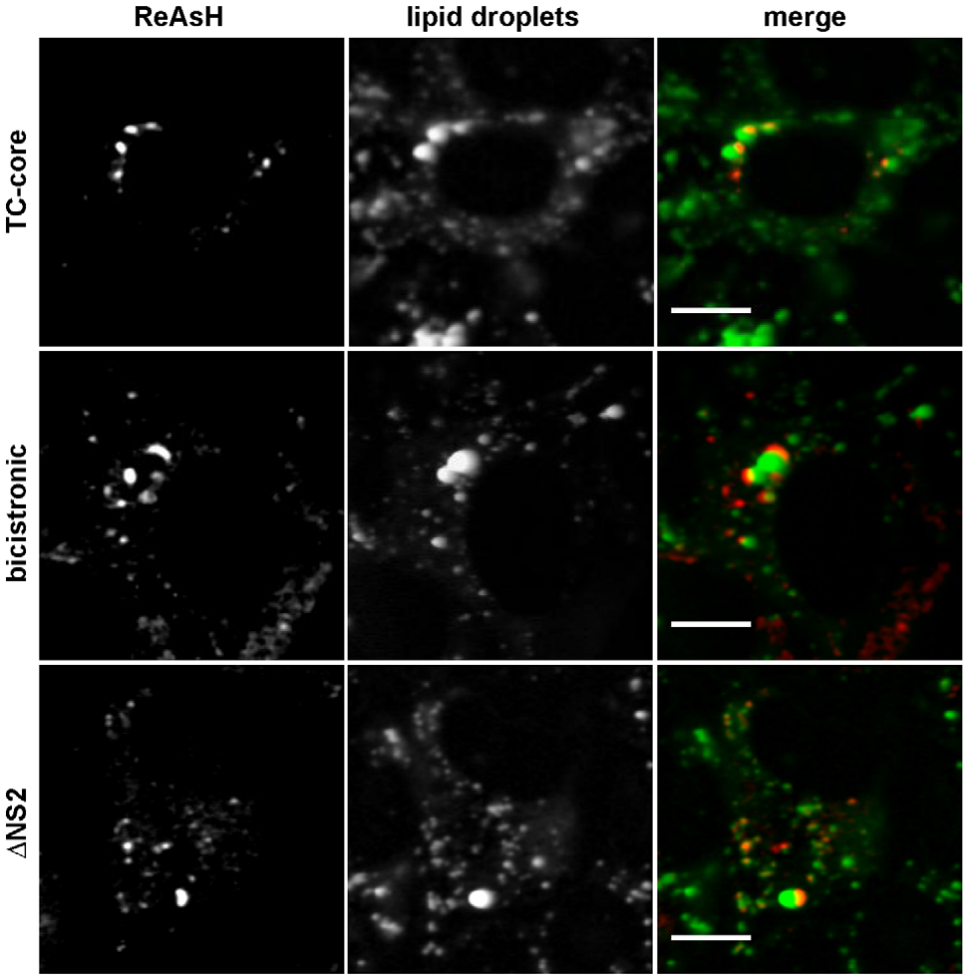
TC-core localizes to lipid droplets. Huh-7.5 cells electroporated with TC-core, bicistronic, or ΔNS2 RNAs were stained with ReAsH dye (red) at 72 hours post electroporation and incubated with Bodipy-493/503 (green) to label lipid droplets. Scale bar is 10 microns.

HCV capsid assembly is dependent on the association of core with the lipid droplet [15]. To examine the association of TC-core with lipid droplets, the various TC-core RNAs were electroporated into Huh-7.5 cells followed by staining with ReAsH dye and the neutral lipid stain (Bodipy 493/503) to detect lipid droplets prior to imaging. All constructs displayed core accumulation in large concentric rings or crescents around larger lipid droplets (Figure 4), indicating that TC-core had appropriate lipid droplet association.

**Figure 5:**
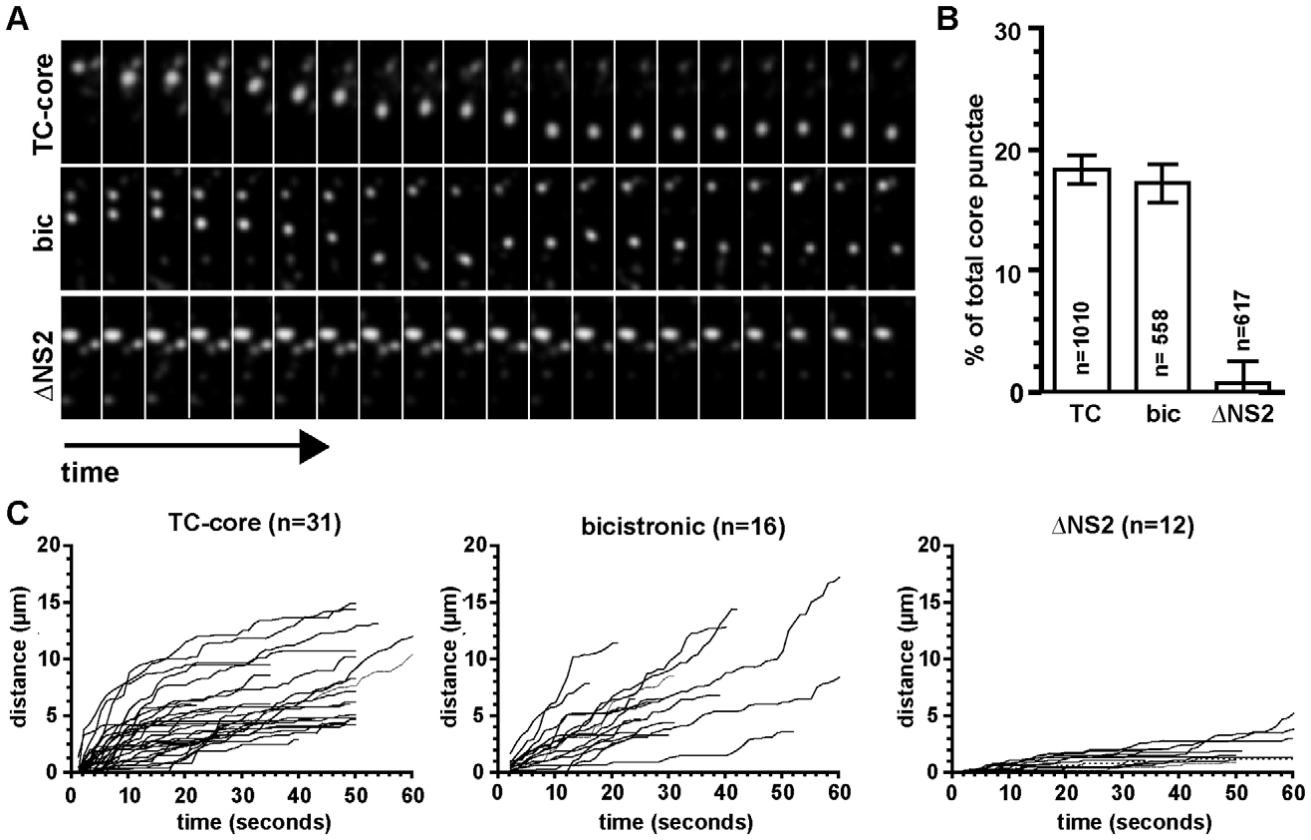
Dynamic TC-core movements require HCV assembly. Huh-7.5 cells were electroporated with TC-core, bicistronic, or ΔNS2 RNAs. Electroporated cells were stained at 72 hours post electroporation with the biarsenical dye ReAsH and imaged using timelapse confocal microscopy where DsRed (200 ms) exposures were taken every 2 seconds. A. Timelapse montages of TC-core puncta transport of the viruses imaged. Time on x-axis. Elapsed times are 40sec (TC-core), 40sec (bicistronic), 132sec (ΔNS2) and height (distance traveled) of each montage is 12.48 µm B. Quantification of percent of TC-core puncta displaying movement over 1 µm during the course of imaging. Values are shown as a percentage of total cellular TC-core puncta. n-values equal total number of TC-core puncta, error bars are standard error (of the proportions). Asterisk indicates *p-value* <0.0001. C. Distance from origin plots of TC-core viruses. The cumulative distance traveled by a TC-core puncta was measured and plotted against time. n-values indicate total TC-core puncta tracked.

We next examined whether the dynamic TC-core movements observed previously (Figure 2C) required HCV virion assembly. Timelapse fluorescence microscopy of individual TC-core puncta was performed 72 hours post electroporation with TC-core, bicistronic or ΔNS2 RNAs. Individual TC-core puncta (of which we can detect using an core antibody) were visualized by live cell confocal microscopy by capturing DsRed exposures every 2 seconds for several minutes (Videos S2–4). Biarsenical dye signal photobleached quickly, therefore timelapse acquisition was limited to short exposures over several minutes (usually up to 10 minutes). A sub-population of cytoplasmic TC-core puncta in cells electroporated with either TC-core or bicistronic RNAs displayed directional movements, whereas ΔNS2 TC-core puncta were static (Figure 5A) or displayed random movements. The TC-core puncta that displayed movements greater than 1 µm as a percentage of total core puncta in a cell was similar between the TC-core and bicistronic populations; 18.2%±1.22, n = 1010 at 72 hours post electroporation (hpe) for TC-core RNAs and 17.2%±1.6, n = 558, for bicistronic RNAs (Figure 5B). In contrast, the frequency of TC-core puncta expressed from the ΔNS2 virus that exhibit directed movement (greater than 1 µm) was virtually nonexistent, 0.65%±1.9, n = 617 (Figure 5B). Extended imaging of the ΔNS2 TC-core virus at 96 and 120 hours post electroporation did not show an increase in TC-core puncta movement (data not shown) indicating cytoplasmic movements of TC-core were dependent on NS2 function independent of viral kinetics.

The dynamics of TC-core trafficking were examined by distance from origin analysis. The cumulative distance traveled (in any direction) by a TC-core puncta during an imaging session was measured and plotted for each virus from a single particle. The distance from origin plots show core puncta in TC-core and bicistronic expressing cells had extensive total movements ranging from 3-17 microns per minute from the point of origin during an imaging session (Figure 5C). Core puncta expressed from the ΔNS2 virus displayed restricted movements ranging from 0-5 microns from the point of origin during imaging (Figure 5A,C), suggesting long range movements occur post assembly.

**Figure 6:**
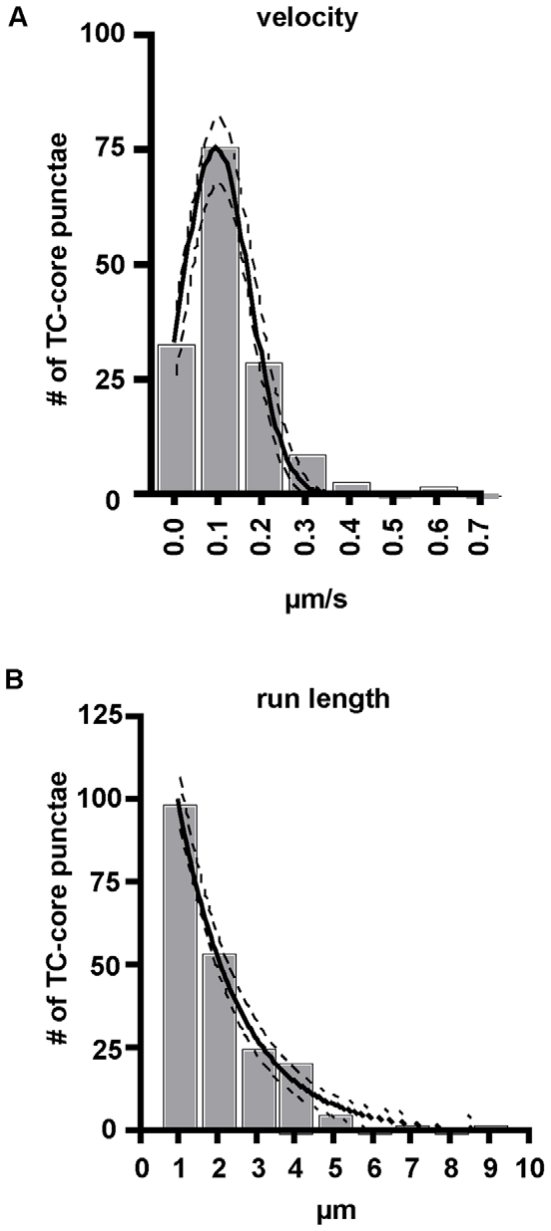
TC-core transport kinetics. Huh7.5 cells were electroporated with TC-core RNA and stained with ReAsH dye at 72 hours post electroporation. Infected cells were imaged by acquiring 200ms exposures every 2 seconds for several minutes. A. Histogram of capsid transport velocities where the x-axis represents velocity in micrometers per second (µm/sec) and the y-axis represents the number of TC-core puncta. The solid curve represents the Gaussian best-fit curve, and the dashed lines indicate the 95% confidence interval. Goodness of fit, *R^2^ = *0.9883. B. Histogram of individual capsid run lengths where the x-axis represents distance traveled in micrometers (µm) ad the y-axis represents the number of TC-core puncta. The solid curve represents the best-fit decaying exponential, and the dashed lines indicate the 95% confidence interval. Goodness of fit, *R^2^ = *0.9924.

The individual run lengths and corresponding velocities of single particles were quantified by manual tracking. A run was defined as a period of uninterrupted transport lacking a pause. A pause was defined as a stop in motion greater than one frame, in this case 2 seconds between image acquisitions. TC-core puncta displayed saltatory (stop and start) movements with few processive runs and reversals where particles switched direction. The average run length displacement of wild type TC-core moving puncta was 2.457 µm ± .1215 (mean ± SEM, n = 154, Figure 6B) and fit a decaying exponential, R^2^ = .9924, which is consistent with microtubule associated transport. The average velocity of moving TC-core puncta was .1336 µm/second ± .010139 (mean ± SEM, n = 154, Figure 6A) and could be modeled by a Gaussian distribution, R^2^ = .9983. The TC-core dynamics of run length and the velocity are consistent with secretory vesicle trafficking (Figure S13) [53], [54].

### TC-core moves in association with microtubules

**Figure 7:**
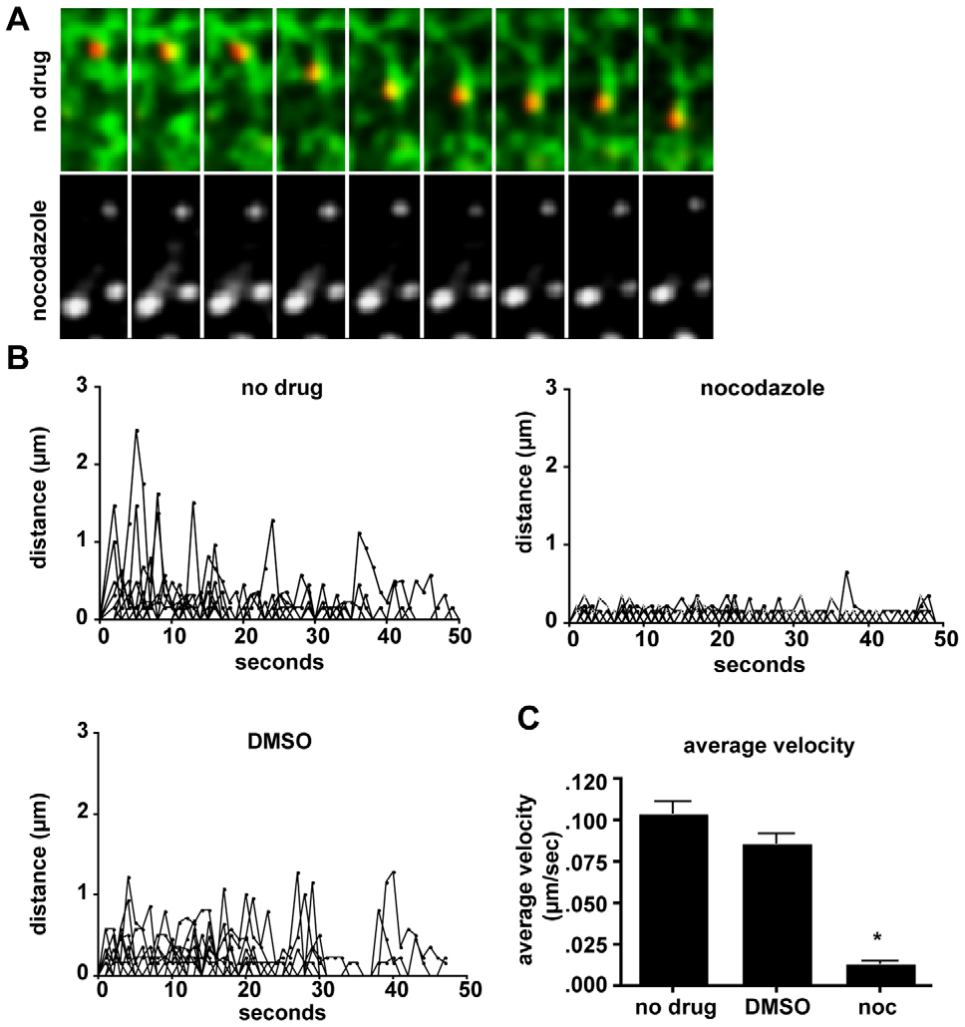
Movement of TC-core is microtubule dependent. Huh-7.5 cells were electroporated with TC-core HCV RNA and at 72 hpe stained with ReAsH (red) followed by incubation with 200 nM TubulinTracker Green. Images were acquired by taking alternating 200 ms DsRed and 200 ms GFP exposures every 2 seconds. A. *Top:* Montage of TC-core (red) transport along microtubule (green). Elapsed time is 1 minute and the length of single frame is 9.76 µm. *Below:* TC-core infected cells stained with ReAsH and TubulinTracker Green and then treated with 8 ug/mL nocodazole immediately prior to imaging. Shown only is TC-core for simplicity, as there is low signal from the depolymerized microtubules. Elapsed time is 2 minutes 18 seconds. The length of the montage is 9.76 µm. B. Single particles from untreated, DMSO, or nocodazole treated cells were tracked and the distance (y-axis) versus time (x-axis) was plotted. Distance plots were constructed using ImageJ Manual Tracking plugin, which provides the instantaneous distance and velocity between two frames. Distance values were plotted versus time (x-axis). C. The average velocities of tracked particles are shown. Error bars represent standard error of mean, asterisk indicates p-value <0.0001.

HCV core mediates redistribution of lipid droplets to the microtubule-organizing center (MTOC) by microtubule directed transport [55]. The dynamics of peripheral TC-core movements are also suggestive of microtubule-dependent transport. To determine if TC-core puncta were associated with microtubules during transport, TC-core electroporated cells were stained with ReAsH at 72hpe and microtubules were visualized with TubulinTracker Green (Invitrogen). Figure 7A (Video S5) shows TC-core puncta movement associated with a microtubule (top). We next tested whether microtubule disruption with nocodazole treatment perturbs TC-core trafficking (Figure 7A-C, Videos S5 and S6). TC-core expressing cells were incubated with nocodazole, DMSO, or left untreated then TC-core puncta dynamics were recorded by timelapse imaging. The run lengths displayed by single particles were determined by manual tracking and the displacement between frames (distance) was plotted against time (Figure 7B). Both the untreated and DMSO treated cells showed similar core dynamics, in which core particles were static, randomly moving, or processively moving. Alternatively, nocodazole treatment dramatically decreased TC-core puncta dynamics (Figure 7A-C), where the majority of particles displayed static or random movements that did not result in long distance displacements.

### TC-core traffics with components of the secretory pathway

**Figure 8:**
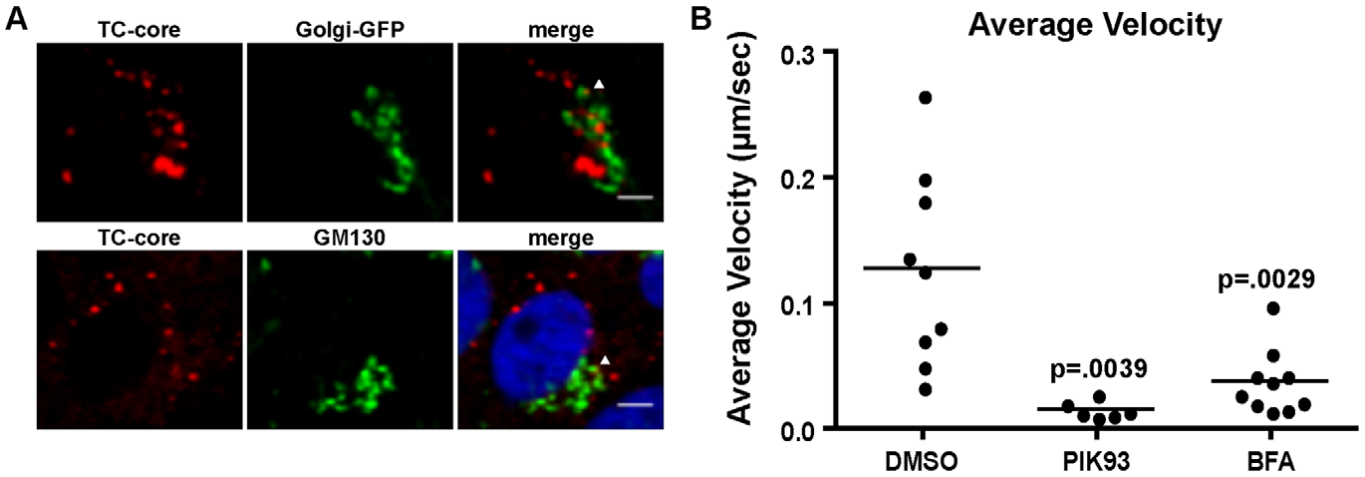
Localization of TC-core and markers of the TGN. A. Huh-7.5 cells were electroporated with TC-core RNA and at 72 hours post infection cells were stained with ReAsH dye. Top panel: Cells were transduced with Golgi-GFP at 48 hours post electroporation then stained with ReAsH. Bottom panels: Cells were stained with ReAsH dye at 72 hours post electroporation and processed for immunofluorescence using an antibody directed against GM130. Arrows point to TC-core puncta colocalized with TGN markers. Scale bar is 10 µm. B. TC-core electroporated cells were incubated with DMSO, PIK93 (0.5 µM), or brefeldin A (BFA) (1 µg/ml) for 2 hours prior to ReAsh staining. Drugs were present in staining and imaging medias. Live cell imaging was performed and TC-core puncta were tracked. The average velocities were measured and are plotted. PIK93 or BFA treatment significantly reduced average velocity (unpaired Student's t-test).

We next validated the results of our RNAi analysis by examining the localization and trafficking of TC-core with cellular cofactors that were identified in the screen. A number of the cellular cofactors are involved in Golgi function and sorting at the TGN, including, CYTH3, PI4KB, PRKD1, and AP1M1. We first examined whether TC-core colocalizes with Golgi markers.Huh-7.5 cells that were electroporated with TC-core RNAs were examined for localization with the Golgi markers Golgi-GFP (Invitrogen, Golgi and Golgi stacks) or GM130 (*cis-* face of the Golgi) (Figure 8). We observe that a sub-population of TC-core colocalizes with the Golgi. The overall frequency of colocalization is relatively low, as would be expected for a transient association of core with the Golgi.

The role of exit from the Golgi in TC-core trafficking was assessed by quantifying the effects on TC-core movements in the presence of inhibitors of two genes identified in the RNAi screen: brefeldin A (BFA), which inhibits ARF3, and PIK93, which inhibits PI4KB. BFA treatment inhibits secretion of viral particles [35]. PI4KB is a resident Golgi lipid kinase that is important in trafficking from the Golgi to the plasma membrane. PIK93 is a selective inhibitor of the type III PI4 kinase, PIK4B, at the concentration used in this experiment (.5 µM) [56], [57]. Treatment with BFA or PIK93 significantly reduced the average velocity of motile TC-core puncta (Figure 8B), further supporting a role of the secretory pathway in HCV secretion.

**Figure 9:**
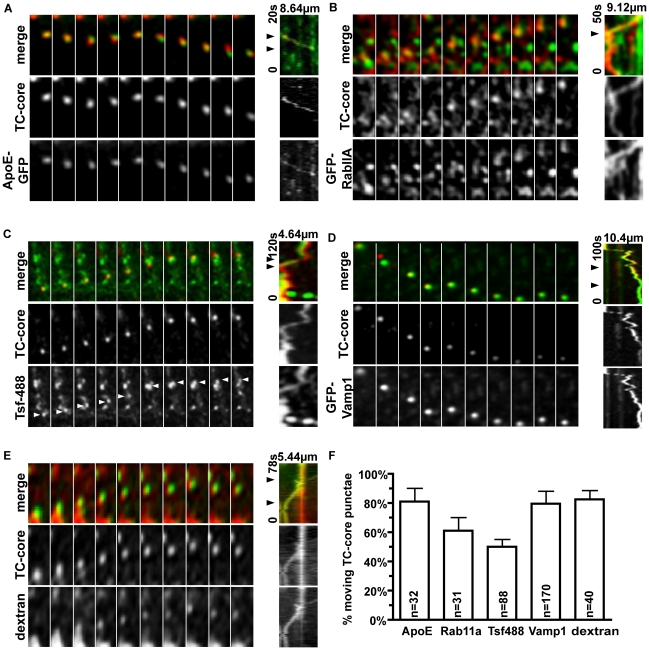
TC-core HCV co-transports with host secretory pathway components. Huh-7.5 cells were electroporated with TC-core RNA followed by transfection at 48 hours post electroporation with either ApoE-GFP (A), GFP-Rab11A (B), or GFP-VAMP1 (D) then ReAsH (red) stained at 72hpe. Otherwise, cells were incubated with transferrin-488 (C) or dextran (E) prior to imaging by confocal microscopy at 72 hours post electroporation. Shown are montages of alternating DsRed (200ms) and EGFP (200ms) exposures taken every 2 seconds. Kymographs (distance on x-axis and time on y-axis) of TC-core and cellular markers are shown to the right of the montage. Straight line on the y-axis indicates stalled transport (time change only), whereas a diagonal line indicates transport (change in time and distance). Distance traveled and time elapsed (seconds) during video acquisition are indicated on the kymograph. Arrows indicate the part of the kymograph shown in montages (left). See supplemental section for videos. F. Quantification of TC-core puncta cotransport with cellular markers as a percentage of total TC-core puncta movers.

We next examined the trafficking of TC-core with ApoE, a component of VLDL secretion [58]. ApoE is required for HCV infection and is associated with extracellular infectious HCV virions [32]–[34], [59], [60]. The association of HCV and lipoproteins is thought to occur early during assembly, then HCV is secreted as a lipo-viroparticle [28], [33], [34], [36], [59], [61]–[63]. In normal hepatocytes, ApoE exits the cell by using the secretory pathway and can also transit through the recycling endosomal pathway [58], [64]–[66]. ApoE is also associated with VLDL particles during secretion [58] and with secretory vesicles leaving the Golgi [66] where VLDL particles mature [67]. We constructed a C-terminal GFP fusion to ApoE, ApoE-GFP, to determine if TC-core puncta traffic with ApoE in live cells. ApoE-GFP had a cytoplasmic localization that could be detected with an ApoE antibody (Figure S4). TC-core expressing cells were transfected with ApoE-GFP at 48hpi followed by live cell imaging at 72hpi. Dynamic TC-core puncta (from transfected and infected cells) were found to colocalize with ApoE-GFP (Figure 9a, Figures S5 & S11, Videos S7 & S13) in the cell periphery. This observation suggests that ApoE is associated with *de novo* forming HCV particles during secretion instead of being acquired extracellularly. We determined the percentage of TC-core puncta moving with ApoE-GFP to be 81.25%±8.98, n = 26/32 of moving TC-core puncta. We also investigated colocalization of core with ApoB. Although some colocalization of core and ApoB was observed in the proximity of lipid droplets, little colocalization between core and ApoB occurred in the periphery (Figure S6).

A gene identified in the RNAi screen to be required for extracellular release of infectious HCV was Rab11A, a small GTP binding protein. Rab11A modulates transport through the recycling endosome [68], associates with apical targeting in polarized cells [69], and is involved in transporting cargoes from the Golgi to the plasma membrane [70]. Rab11 is required for budding of influenza virus [71], hantavirus release [72], and involved in the vesicular trafficking of Sendai virus ribonucleocapsids [73]. We constructed a N-terminal GFP fusion to Rab11A, and imaged TC-core and Rab11A cotrafficking by timelapse fluorescence microscopy (Figure 9B, Figures S4 & S7, Videos S8 and S14). GFP-Rab11a had a cytoplasmic localization that was either associated with the Golgi network or alone in puncta (Figure S4). We also visualized TC-core with a second marker of the recycling endosome, transferrin. TC-core electroporated Huh-7.5 cells were incubated with Alexa Fluor 488 conjugated transferrin, and cotrafficking of TC-core and transferrin was imaged using live cell microscopy (Figure 9C, Figure S8, Video S9). We determined the percentage of moving TC-core puncta associated with Rab11a and transferrin 488 markers to be 61.2%±8.7% (n = 19/31 of moving TC-core puncta) and 50.0%±5.3% (n = 44/88 of moving TC-core puncta), respectively. The cotrafficking of TC-core with markers of the recycling endosome validates the RNAi data that implicates a role for the recycling endosome in infectious HCV secretion.

**Figure 10:**
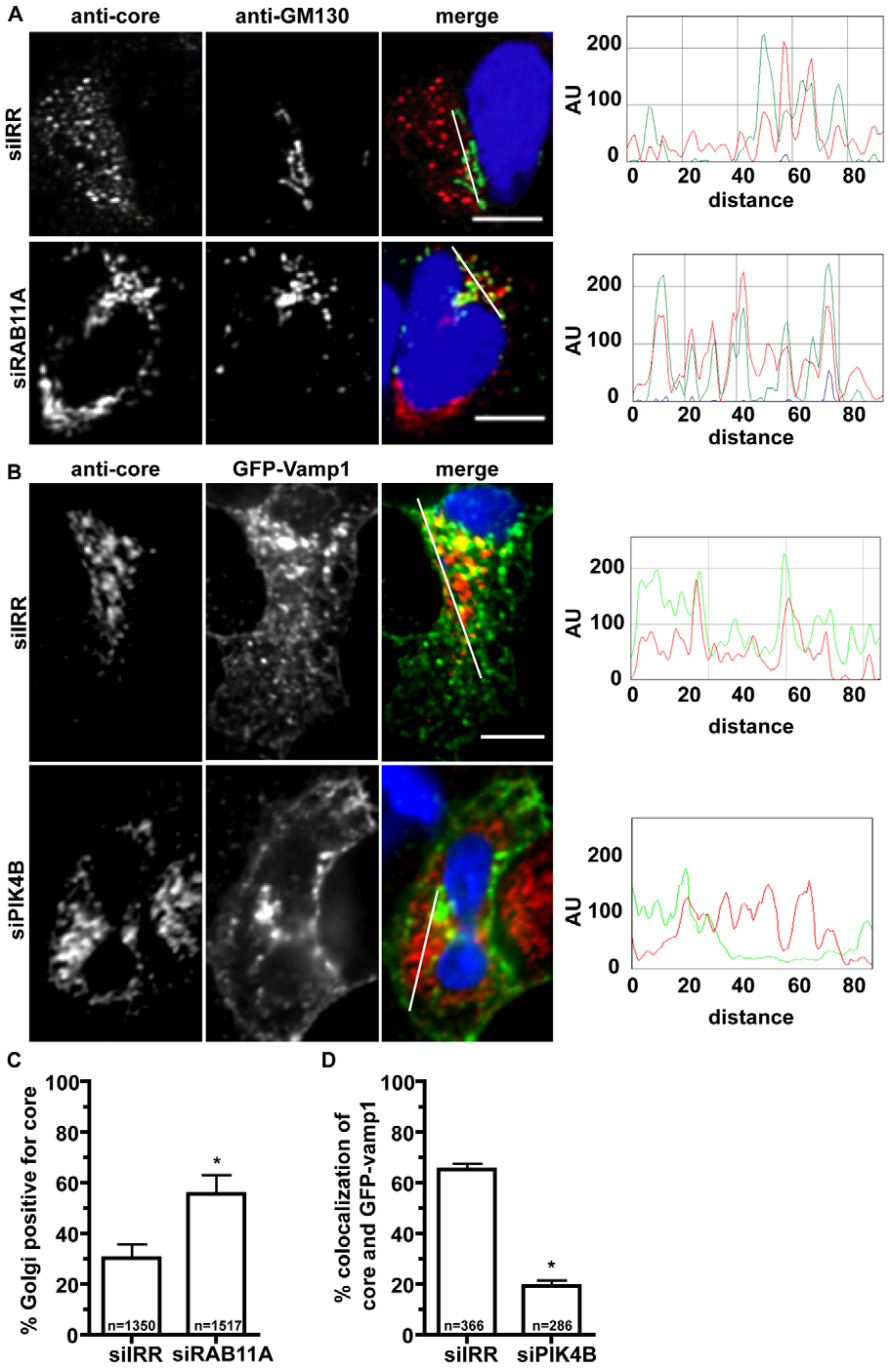
siRNA treatments inhibiting infectious virus production alter core sub-cellular localization. A. Huh7.5 cells were electroporated with irrelevant (siIRR) or Rab11a (siRab11a) siRNAs followed by infection with HCV at 48 hours post electroporation. Cells were fixed and stained with Golgi marker GM130 (green) and core (red) at 72 hours post infection. Immunofluorescence of siIRR (top) and siRab11a (bottom) treated cells expressing viral core. White line in merge image is profiled for fluorescence intensity. RBG profile where distance is in pixels and fluorescence intensity is in arbitrary fluorescence units (AU). Scale bar is 10 µm. B. Huh-7.5 cells electroporated with irrelevant (siIRR) or Pik4B (siPIK4B) siRNAs, then infected with HCV at 48hpe followed by transfection with GFP-Vamp1 (green) at 72hpe. Cells were maintained for one day, then fixation and stained with anti-core antibody (red). Top: siIRR treatment. Bottom. si-PI4KB treatment. RBG profile where distance is in pixels and fluorescence intensity is in arbitrary fluorescence units (AU). Scale bar is 10 µm. C. Quantitation of core-GM130 colocalization in (A). Asterisk indicates p-value <.01. D. Quantitation of core Vamp1-GFP colocalization in (B). Asterisk indicates p-value <.0001.

Although the cotrafficking of TC-core with various cellular markers was initially done in cells electroporated with TC-core HCV RNAs, we subsequently confirmed co-trafficking the results following infection with TC-core virus (Figure S11). We next tested whether the defects in infectious HCV production following Rab11a silencing were associated with altered core trafficking. Huh-7.5 cells were electroporated with Rab11a siRNAs, maintained for two days, infected with HCV for two days, fixed and probed with antibodies to core and the Golgi marker GM130. We observe an increase in core accumulation at the Golgi following Rab11a silencing (Figure 10A,C, 30.2±5.5% (n = 1350)) of Golgi stacks are core positive in siIRR treated cells versus 55.7±7.4% (n = 1517) in siRab11a treated cells. Thus, the defect in infectious HCV release in siRab11a treated cells correlates with an accumulation of core in the Golgi network.

Vesicular associated membrane 1 (VAMP1) was identified as a cofactor in the release of extracellular infectious HCV in our RNAi screen. VAMP1 can complex with syntaxins and SNAP25 to form a SNARE complex involved in docking and/or fusion of vesicles at the plasma membrane [74]. We constructed a N-terminal GFP fusion to VAMP1 and visualized cotransport of GFP-VAMP1 and TC-core (Figure 9D, Figure S9, Video S10 and S15). GFP-VAMP1 localized to puncta in the cytoplasm and could be detected using an anti-VAMP1 antibody (Figure S4). We found a high percentage of TC-core puncta (79.4% ± 8.90 n = 135/170 of moving TC-core puncta) co-trafficking with GFP-VAMP1 in the cell periphery suggesting that TC-core is transported in VAMP1 associated vesicles. It is well established that the secretory and endosomal pathways intersect to deliver vesicular cargoes to the plasma membrane. To determine if TC-core puncta transported in the lumen of a vesicle, we incubated TC-core electroporated cells with dextran and found that the dynamic TC-core trafficked with dextran (Figure 9E, Figure S10, Supplemental Video S11) with a frequency of 82.5%± 6.0% (n = 33/40 moving TC-core puncta). This evidence further supports that core is vesicle-associated during secretion.

Finally, we tested whether silencing the Golgi lipid kinase PI4KB would decrease the association of core with VAMP1. siRNA treated cells were transfected with GFP-VAMP1, infected with wild type HCV for two days and processed for core and GFP-VAMP1 imaging. We observed extensive colocalization of core and GFP-VAMP1 (65.1±2.5% (n = 366)) in siIRR treated cells (Figure 10B and D). However in cells treated with siPIK4B, fewer core and GFP-VAMP1 colocalization (19.2±2.3% (n = 286)) was observed (Figure 10B and D). Thus, PI4KB expression is required for core colocalization with VAMP1.

## Discussion

This study combined a RNAi analysis to identify host genes involved in the release of infectious HCV from cells with live cell imaging of HCV core trafficking in order to assess the dynamics and cellular pathways involved in HCV egress. The data from the RNAi screen delineates an egress pathway for HCV that heavily interplays with the constitutive secretory pathway, which is suggested for other viruses in the *Flaviviridae* (reviewed in [75]). If we align the cellular cofactors of HCV egress along the constitutive secretory pathway and also supplement with data from the literature, the following molecular model of infectious HCV secretion in Huh-7.5 cells is attained. HCV is hypothesized to initiate assembly in close association with lipid droplets by coating lipid droplets with the core protein and bringing together nonstructural and structural proteins in a NS2-dependent manner [23], [51], [76]. Following capsid assembly, nascent virions bud into the lumen of the ER where the glycoproteins E1/E2 reside in addition to the VLDL secretion machinery. HCV is infectious upon envelopment at the ER, and it is thought that ApoE is acquired early during assembly because knockdown of ApoE reduces intracellular and extracellular virus [32]; also NS5A interacts with ApoE [59], [63]. We found through live cell imaging TC-core cotransports extensively with ApoE-GFP (Figure 9A). In addition, ApoE localizes to large, static, crescent shaped accumulations of core presumably associated with lipid droplets (Figure S12). Our data does not clearly distinguish whether HCV hijacks the intact VLDL secretion pathway or associates with ApoE through the NS5A interaction; however, the absence of ApoB colocalization with TC-core puncta is more supportive of the latter model (Figure S6).

The maturation of HCV particles overlaps with other aspects of VLDL secretion. Nascent VLDL particles undergo further lipidation and maturation in the Golgi [77], [78] followed by ER to Golgi trafficking dependent on SAR1A [67]. Our RNAi screen implicated SAR1A in the production of extracellular HCV suggesting HCV particles transport from the ER to the Golgi with COPII secretory vesicles. The Golgi is further associated with HCV secretion by the genes CYTH3, ARF3, AP1M1, PI4KB, and PRKD1. During secretion, ARF GTPases such as CYTH3 and ARF3 are activated then localize to the TGN where they can interact with coat proteins, such as AP1M1 to initiate vesicle budding and sorting (reviewed in [79]). Treatment of HCV infected cells with brefeldin A, which can inhibit ARF3, results in the inhibition of infectious extracellular virus release [35]. We visualized association of TC-core particles with TGN markers. In contrast to a recent study [80], we did not find evidence of Golgi disruption in either wild type HCV or TC-core (Figure 8) infected cells and we found PRKD1 to be required for HCV release. PI4KB (phosphatidylinositol kinase IIIβ) appears to influence the replication of some HCV genotype 1 isolates [81], [82]. We observe that in the case of J6/JFH1 infection, it plays a role in egress, and not in entry or replication [44]. We cannot discount the possibility that some of the siRNA treatments may affect secondary functions, such as the post-translational modifications of viral proteins.

Following maturation of particles in the TGN, cargoes are sorted into secretory vesicles en route to the plasma membrane. Rab GTPases are master regulators of intracellular vesicle trafficking, and we found that Rabs 11A and 3D were required for efficient secretion of HCV. Rab11A positive recycling endosomes sort some vesicles during exocytosis (reviewed in [83]) to apical and basolateral membranes. We found TC-core puncta colocalized with GFP-Rab11A and transferrin-488, a marker for the recycling endosome, and some of these puncta displayed directional transport (Figure 9B). The transient nature of the interaction may be attributed to the recycling endosome serving as a sorting station for membrane bound cargoes. Silencing Rab11a leads to the increased accumulation of core in the Golgi, further supporting a model wherein HCV virions bud from the TGN and are transported to the recycling endosome.

VAMP1 and Rab11A were implicated in ours and other [84] RNAi screens for host factors involved in HCV propagation. VAMP1 is a v-snare that is loaded onto secretory vesicles at the TGN and mediates the fusion of the vesicle with the plasma membrane by interacting with SNAP-25 and syntaxins [74]. We found a dynamic sub-population of TC-core that transports long-range distances with GFP-VAMP1. TC-core also cotransports with dextran, which also indicates vesicle association. Interestingly, HCV core has been shown to localize with early and late endosomal markers during egress time points and the release of extracellular virus depended on endosome motility [85]. We have confirmed that a smaller sub-population of TC-core cotraffics with Rab5a-GFP labeled early endosomes (data not shown), however, the interpretation of this data is complicated by the core-rab5A interactions observed during endocytosis [43]. Due to the design of the screen (excluding genes involved in HCVpp entry or replicon replication), it is possible that some genes with multiple roles in the viral life cycle were overlooked.

Trafficking of secretory vesicles and TC-core both require intact microtubules (Figure 7). This is also supported by the observation that the release of infectious extracellular HCV is disrupted by depolymerization of microtubules [55]. We also found genes involved in actin dynamics (RHOA, GIT1, WAS) to play a role in HCV secretion. It is not thought actin dynamics are responsible for the long-range transport we recorded for the TC-core virus, but actin may play a role in the biogenesis of transport vesicles (reviewed in [86]) and fusion of secretory vesicles at the plasma membrane.

The dynamics of TC-core movements are consistent with vesicular trafficking in the Huh-7.5 cells (Figure S13). HCV particles, especially as lipo-viroparticles, are too large to freely diffuse through the cytoplasm; instead they likely rely on microtubules for long-range transport. Secreted HCV particles may have several associated microtubule motors because TC-core puncta display bidirectional transport. Because the majority of TC-core puncta moving towards the plasma membrane (plus-end transport) displayed a velocity curve that could be modeled by a Gaussian distribution, a single class of plus ended motors is most likely involved. Association of a minus-end motor is likely since particles displayed bidirectionality, but we cannot rule out mixed microtubule polarity in our Huh-7.5 cells.

Transport rates for TC-core puncta are below what has been published for other viruses. Neurotropic herpesviruses display transport kinetics consistent with fast axonal transport (2 µm/sec), which does not occur in hepatocytes, and vaccinia virus displays slower saltatory movements (1-3 µm/sec) (reviewed in [87]). In our study, we found HCV core to move at rates similar to Vamp-1 and ApoE-associated vesicles in Huh-7.5 cells (Figure S13). Long-range transport to the plasma membrane represented a net plus-end movement that is comprised of small steps along the microtubule. The slow rates of TC-core movement that we recorded can be better resolved with higher spatial and temporal resolutions, as we were only imaging rates of 1 frame per 2 seconds.

Previous characterization of HCV JC1 infection found less core localization at lipid droplets and more ER localization than is observed in HCV JFH1 infection [88]. This was interpreted as possibly reflecting differences in the sites of assembly for HCV JC1 and JFH1. In our experiments, the TC-core from JC1 clearly localizes at lipid droplets, in addition to the ER, Golgi, and other secretory components. The predominant subcellular localization of core that is observed in fixed cell microscopy does not necessarily indicate a site of virion assembly, it simply reflects which stage in core trafficking is rate limiting. For instance, a slightly different interpretation is that JFH1 assembles virus at a lower efficiency than JC1, therefore more core is at the lipid droplet awaiting completion of virion assembly. Since JC1 virion assembly is more efficient than JFH1, more core is localized to other subcellular compartments involved in core trafficking, such as the ER. In support of this interpretation, our TC-core virus has a higher amount of lipid droplet localization than the parental JC1 due to slightly less efficient virion assembly.

We plan to revisit the trafficking studies in a more relevant polarized hepatocyte cell culture system. Hepatocytes *in vivo* possess complex architecture that consists of distinct basolateral (sinusoidal) and apical (canalicular) plasma membrane domains, which are separated by tight junctions. The association of apical membranes from neighboring cells forms bile caniculi. With complex polarity, also comes complex trafficking of proteins to apical and basolateral membranes. Two pathways are used to direct proteins to the apical membrane in hepatocytes, a direct route or through transcytosis after reaching the basolateral membrane. HCV is known to spread via two distinct routes, extra-cellular release and cell-cell spread [89], [90]. Future studies will characterize the cell-cell spread trafficking pathway and examine whether HCV undergoes selective polarized trafficking to ultimately spread through an extracellular (basolateral) or intracellular (apical) intermediate.

## Materials and Methods

### Virus and cells

Huh-7.5 cells were grown in Dulbecco's modified high glucose media (DMEM; Invitrogen) supplemented with 10% fetal bovine serum (FBS; Invitrogen), nonessential amino acids (NEAA, 0.1 mM; Gibco), and 1% penicillin-streptomycin (Invitrogen). Viral RNA was electroporated into Huh-7.5 cells as described previously [43], [44]. Viral supernatants were collected daily from up to 5 passages of electroporated cells, filtered through a .22 µm nitrocellulose filter, and kept at 4°C protected from light.

### Plasmid constructs

pJFHxJ6-CNS2C3 (WT) is a HCV genotype 2a infectious clone where core through the first transmembrane domain of NS2 of a JFH-1 backbone was replaced with J6 genotype sequence [91]. A full length (FLN**CCPGCC**MEP) tetracysteine (TC) tag [92] was inserted into the HCV core gene after amino acid 3 by amplifying two regions (5′ and 3′ of the tag) and joining the two regions with an ApaLI restriction site proximal to the tag insertion site. The full length TC tag has been shown to enhance binding of biarsenical dyes [92]. The 5′ region was amplified using 5′ AACGAATTCTAATACGACTCACTATAGACC and 5′ AACGTGCACGGTCTACGAGACCTCCCGGGG (*EcoR*I and *ApaL*I sites underlined). The 3′ region was amplified using 5′ AACGTGCACCATGAGCACA**TTTCTCAATTGTTGTCCTGGCTGTTGTATGGAACCT**AATCCTAAACCTCAAAGAAAAACC and 5′ AACCGTACG CCAAGATCATGGTAGCCGTGG (*ApaL*I and *BsiW*I sites underlined, TC tag bolded). The 5′ and 3′ regions were digested with ApaLI and ligated together to form the full-length core region with the tag inserted. The TC-core fragment was subsequently cloned into JFHxJ6NS23 using the *EcoR*I and *BsiW*I sites and the TC tag insertion was confirmed by sequencing. The bicistronic construct was constructed by insertion of a HCV genotype 2a IRES – NS2 fragment into a subgenomic replicon, pSGR-JFH1-neo, containing the encephalomyocarditis virus (EMCV) IRES through the HCV replicase genes NS3-NS5B [52]. The IRES-NS2 fragment was PCR amplified by using the primers 5′ACCGAATTCTAATACGACTCACTATAGACCTGCCCCTAATAG and 5′ ACCGTTTAAACTTAAAGGAGCTTCCACCCCTTGGAGGTGTAGCCATCAGCTGG (*EcoR*I and *Pme*I sites are underlined). The subsequent fragment was subcloned in the replicon resulting in the bicistronic construct. The NS2 deletion construct was made by two-part subclone. NS2 was deleted by insertion of the HCV genotype 2a IRES-p7 genes into a subgenomic replicon as stated above. The IRES-p7 region was PCR amplified by using the same *EcoR*I primer as above and 5′ ACCGTTTAAACTTAAGCATAAGCCTGTTGGGGCAATGCTAGGAGCAGTAGGCTA (*Pme*I site underlined) which goes through p7. The resulting PCR fragment was cloned in the subgenomic replicon stated above. This resulted in the NS2 deletion virus in a bicistronic backbone. The TC-tagged core was introduced by digestion of the TC-core virus with *EcoR*I and *BsiW*I to liberate a DNA fragment containing TC-core through part of glycoprotein E1. Untagged core in the NS2 deletion backbone was replaced with the TC-core fragment using the corresponding *EcoR*I and *BsiW*I restriction sites. This resulted in a TC-core NS2 deletion (ΔNS2) construct. The resulting clone was sequenced to verify in frame insertion of the tag and presence of the deletion.

Apolipoprotein E was PCR amplified from a human ORF clone, accession number EL733202 (Open Biosystems), using the primers 5′ ACGAATTCATGAAGGTTCTGTGGGCTGC (*EcoR*I site is underlined) and 5′ GTGGATCC **C**GTGATTGTCGCTGGGCAC (*Bam*HI site is underlined, bold indicates a 1nt insertion). The PCR product was cloned into pEGFP-N2 at the *EcoR*I and *Bam*HI sites to create ApoE-GFP, and then confirmed by sequencing. Rab11A was PCR amplified from a human ORF clone, accession number CV027643 (Open Biosystems), using the primers 5′ ACCAAGCTTCGATGGGCACCCGCGACGACGAGTACGAC (*Hind*III site is underlined) and 5′ ACCGGTACCTTAGATGTTCTGACAGCACTGCACCTT (*Kpn*I site is underlined). The PCR product was cloned into pmGFP-C1, resulting in GFP-Rab11A and confirmed by sequencing. VAMP1 ORF was PCR amplified from a human ORF clone, accession number CV029376 (Open Biosystems), using the primers 5′ GCAGATCT ATGTCTGCTCCAGCTCAGCCACC (*Bgl*II site is underlined) and 5′ GCAAGCTT TCAGCGATACTTACTTACAATAAC (*Hind*III site is underlined). The resulting PCR product was cloned into pEGFP-C1 using the *Bgl*II and *Hind*III sites to result in GFP-VAMP1 and confirmed by sequencing.

### Viral replication curve and TCID50/mL

WT, TC-core, bicistronic, and ΔNS2 RNAs were transcribed *in vitro* as described [52]. Huh-7.5 cells were electroporated with 2 µg/µl of each viral RNA and plated into 96 well plates. Viral supernatants were collected at indicated times post electroporation and titered by limiting dilution and immunohistochemical staining using an antibody directed to NS5A (9E10) as described [1], [93]. For intracellular infectious virus, the cells went through three freeze-thaw cycles prior to titering. For RNA quantitation, cells were washed twice with PBS and lysed in RLT lysis buffer (Qiagen) at indicated timepoints. RNA was isolated using the RNAeasy kit (Qiagen). RNA copy number was determined by reverse transcriptase (RT) PCR analysis as described previously [43], [44] using the Platinum qRT-PCR Thermoscript One-Step System (Applied Biosystems). 2 µL of extracted RNA was detected using the following primers and probe: forward 5′ ACTTCATTAGCGGCATCCAATAC; reverse 5′-CGGCACTGAATGCCATCAT; probe 5′-6FAM-CAGGATTGTCAACACTGCCAGGGAACC - (Iowa Black), amplifies from the NS4 gene. PCR conditions were 50°C for 30 minutes, 95°C for 6 minutes, and (95°C for 15 seconds followed by 60°C for 1 minute) x 50 cycles.

### RNA interference assay

The siRNAs used are listed in Table S1. The methods for the primary RNAi screen have been previously described [43], [44]. Briefly, the primary screen included pools of four siRNAs that target 140 membrane trafficking genes (Dharmacon, Inc.). Genes important for infectious HCV production were subsequently silenced with four individual siRNAs targeting the gene to validate the RNAi results. 1×10^6^ Huh-7.5 cells in 0.05 ml of PBS pH 7.4 were electroporated with 125 picomoles of siRNA for 5 pulses of 770 volts for 99 microseconds with one- second intervals on a BTX 830 electroporator with 96-well attachment. Cells were infected 72 hours after electroporation with a multiplicity of 0.5 infectious HCV particles per cell for 6 hours, rinsed with media, then maintained for 2 days at 37°C. 48 hours after infection, the supernatants were collected and analyzed for infectious virus via limiting titer dilution as described previously [93]. Host gene expression knockdown was assessed using the primer – probe sets listed in Table S2.

For visualization of core localization during silencing, cells were silenced with siRNAs followed by infection and immunofluorescence. Briefly, Huh-7.5 cells were electroporated (as above) with 2 pre-validated RAB11a siRNAs (Ambion, ID: s16073 and 14940), 2 pre-validated PIK4B siRNAs (Ambion, ID: 283 and s10543), or irrelevant siRNA and silencing was established for 48 hours. Electroporated cells were infected with HCV then maintained for 2 days, followed by seeding onto coverslips, fixation, and immunofluorescence staining.

### Immunofluorescence

HCV TC-core infected cells stained with biarsenical dye were fixed in 4% paraformaldehyde for 10 min, permeabilized in 0.2% Triton X-100 in PBS for 20 minutes, and blocked with 0.1% Tween-20 and 10% goat serum in PBS for 30 min. Cell were washed with PBS plus 0.1% Tween-20 (PBST) after blocking and between antibody incubations. Primary and secondary antibodies were diluted in PBST. For detection of core, a mouse monoclonal antibody (#1851 Virostat, Portland, ME) was used at a dilution of 1∶50, mouse monoclonal antibody to NS5A (9E10) was used at a dilution of 1∶1000, dsRNA antibody (J2, English and Scientific Consulting Bt) was used at 1∶1000, and a human monoclonal antibody to E2 (Steve Foung) was used at 1∶250. GFP fusion proteins were assessed for correct localization by incubation with antibodies. Anti-Apolipoprotein E (Abcam) was used at a dilution of 1∶250, anti-Vamp1 (Abcam) was used at a dilution of 1∶250, anti-Rab11a (Invitrogen) was used at a dilution of 1∶250, anti-GM130 (Abcam) was used at 1∶250 to label the Golgi network, and anti-ApoB was used at a dilution of 1∶250. Alexa-488 or Alexa-594 conjugated IgG (Molecular Probes, Eugene, OR) secondary antibodies were diluted 1∶1000. Golgi-GFP (Invitrogen) was used to label the TGN by the recommended directions. Bodipy 493/503 was used to label neutral lipids (lipid droplets) in both fixed and live cell images at a concentration of 20 µg/ml.

### Biarsenical dye labeling of HCV expressing cells

FlAsH or ReAsH labeling was performed per the instructions provided in the TC-tag detection kit (Invitrogen). At 72 hours post electroporation, cells were washed once with Opti-MEM (Invitrogen) and labeled with biarsenical dye (1.25 µM) in Opti-MEM. Cells were incubated at 37°C for 30 minutes, then washed two times with 1X BAL (2,3-dimercapto-1-propanol) wash buffer (supplied in the kit, Invitrogen) supplemented with 500 µM EDT in Opti-MEM for 5 minutes. The wash buffer was removed and the cells were washed one time using Opti-MEM followed by incubation with prewarmed media.

Tracking of TC-core in the presence of drug inhibitors of the secretory pathway was performed as follows: TC-core electroporated cells were incubated with DMSO, GolgiPlug containing BFA (1 µg/mL, BD Biosciences), or PIK93 (0.5 µM, Tocris) for 2 hours prior to staining with ReAsh. DMSO or drug was present during time of staining and in the imaging media.

### RNA labeling

Infected cells were incubated with Click-iT RNA Alexa Fluor 488 Imaging Kit (Invitrogen) to visualize RNA. Briefly, infected cells were seeded on round coverslips and incubated with Click-iT kit as per manufacturer's instructions in the presence of actinomycin D (1 µg/ml). Cells were processed for immunofluoresence after Click-iT labeling.

### Fluorescence microscopy

All images were acquired with an Olympus DSU spinning disk confocal microscope fitted with a 100×1.45 N.A oil-immersion objective. FlAsH labeled core was detected using the EGFP filter set (ex: 480/25 and em: 525/40), whereas ReAsH labeled core was visualized with the DsRed filter set (ex: 565/25 and em: 620/60) coupled to a Hamamatsu back-thinned EM-CCD high speed/sensitivity camera. Slidebook software was used for image acquisition and processing (Intelligent Imaging Innovations, Inc, Denver, CO).

Immunofluorescence imaging of TC-core with viral or host markers was accomplished by acquiring sequential static images with DsRed, EGFP, and 350 filter set sequential exposures.

Live cell imaging of TC tagged HCV was performed by growing infected cells on polylysine treated 35 mm glass bottom Fluorodishes (World Precision Instruments). Cells were stained with biarsenical dye as above except imaging media (DMEM-F12- Invitrogen) supplemented with 10% fetal bovine serum, 0.1 mM nonessential amino acids, 1% penicillin-streptomycin, and 25 mM HEPES was added following the final wash. Imaging chambers were sealed with parafilm before imaging. Cells were maintained on a heated stage set at 37°C. Timelapse imaging of TC-core particles and GFP labeled host proteins was taken by sequential imaging every 2 seconds with 200 ms exposures for each channel, DsRed and GFP respectively. In some cases cells were incubated with transferrin-488 (5 µg/mL, Invitrogen) or phrododextran (10 µg/mL, Invitrogen) prior to imaging as per manufacturer's instructions.

Individual core puncta transport velocities and run lengths were calculated by kymograph analysis. Briefly, the multiple kymograph plugin for ImageJ was used to generate kymographs. Runs were defined as uninterrupted diagonal lines and the distance traveled (length) and the average velocity (slope) were measured. The frequency of TC-core puncta movement was measured by quantifying the number of TC-core puncta displaying a run length greater than 1 µm as a percentage of total cellular TC-core puncta signal using the analyze particles tool in ImageJ per imaging session. Distance from origin plots were generated by using the Manual Tracking plugin for Image J, measuring the distance traveled (in any direction) between frames for a respective TC-core puncta. Values were added and plotted against time. Manual Tracking plugin was also used to generate the distance versus time plots. Distance between timepoints was plotted versus time.

### Statistical analysis

Prism software was used to plot and determine *p-values* for replication and TCID50/mL titer curves. The velocity and run length plots were also done using Prism software where the data was modeled to a Gaussian distribution or a decaying exponential.

## Supporting Information

Figure S1 **Cell viability following electroporation with the indicated siRNAs.** Viability of siRNA-treated cells was measured at 5 days post electroporation in two distinct experiments (A) and (B) by a luminescence-based assay (Promega) that measures intracellular ATP levels. SEM is shown.
(TIF)

Figure S2 **Intra- and extra-cellular infectious virus following siRNA treatment.** Relative levels of intra-and extra-cellular infectious virus levels from Table 1.
(TIF)

Figure S3 **Biarsenical dyes do not affect virus release.** Huh-7.5 cells were infected with wildtype virus for 48 hours then incubated with ReAsh dye for 30 minutes. ReAsh dye was removed and cells were washed with 1x BAL buffer supplemented with 500 µM EDT. Cells were incubated in fresh media and supernatants were collect at 2, 8, 24, 48 hours post ReAsh incubation and titered.
(TIF)

Figure S4 **Immunofluorescence of GFP fusions of ApoE, Vamp1, and Rab11a.** Huh7.5 cells were transfected with ApoE-GFP, GFP-Vamp1, or GFP-Rab11a. Cells were fixed and processed for immunofluorescence using antibodies directed against ApoE (top), Rab11a (middle), GM130 (middle bottom) or Vamp1 (bottom) and corresponding secondary antibodies (red). Scale bar is 10 µm.
(TIF)

Figure S5 **TC-core cotransports with ApoE-GFP.** Huh-7.5 cells were electroporated with TC-core RNA and transfected with ApoE-GFP at 48 hpe. Cells were stained with ReAsh (red) at 72 hpe then imaged. Boxed region contains TC-core puncta shown in montage in Figure 9A and as a Video S7. Line indicates trajectory of moving particle. Scale bar  =  10 µm.
(TIF)

Figure S6 **ApoB does not colocalize with TC-core puncta.** Huh7.5 cells were infected with TC-core virus and stained with ReAsh dye (red) at 72 hours post infection followed by processing for immunofluorescence using an ApoB antibody (green). Scale bar is 10 µm.
(TIF)

Figure S7 **TC-core cotransports with GFP-Rab11a.** Huh-7.5 cells were electroporated with TC-core RNA and transfected with GFP-Rab11a at 48 hpe. Cells were stained with ReAsh (red) at 72 hpe then imaged. Boxed region contains TC-core puncta shown in montage in Figure 9B and as a Video S8. Line indicates trajectory of moving particle. Scale bar  =  10 µm.
(TIF)

Figure S8 **TC-core cotransports with Alexa Fluor 488 transferrin.** Huh-7.5 cells were electroporated with TC-core RNA and stained with ReAsh (red) at 72 hpe then incubated with Alexa Fluor 488 transferrin (green). Cells were immediately imaged. Boxed region contains TC-core puncta shown in montage in Figure 9C and as a Video S9. Line indicates trajectory of moving particle. Scale bar  =  10 µm.
(TIF)

Figure S9 **TC-core cotransports with GFP-VAMP1.** Huh-7.5 cells were electroporated with TC-core RNA and transfected with GFP-VAMP1 at 48 hpe. Cells were stained with ReAsh (red) at 72 hpe then imaged. Boxed region contains TC-core puncta shown in montage in Figure 9D and as a Video S10. Line indicates trajectory of moving particle. Scale bar  =  10 µm.
(TIF)

Figure S10 **TC-core cotransports with dextran.** Huh-7.5 cells were electroporated with TC-core RNA and stained with FlAsh (green) at 72 hpe then incubated with dextran (red). Cells were immediately imaged. Boxed region contains TC-core puncta shown in montage in Figure 9E and as a Video S11. Line indicates trajectory of moving particle. Scale bar  =  10 µm.
(TIF)

Figure S11 **TC-core produced in infected cells co-transports with host secretory pathway components.** Huh-7.5 cells were infected with TC-core virus followed by transfection with either ApoE-GFP (A), GFP-Rab11a (B), or GFP-Vamp1 (C) plasmids at 24 hours post infection (hpi). Cells were incubated with ReAsh at 72hpi followed by live cell confocal microscopy. Shown are time-lapse montages of alternating DsRed (200ms) and EGFP (200ms) exposures taken every 2 seconds for several minutes. Kymographs are shown to the right of each montage.
(TIF)

Figure S12 **ApoE-TC-core colocalization at core accumulations.** Huh-7.5 cells were electroporated with TC-core RNA and transfected with ApoE-GFP at 48 hours post electroporation. Shown is ApoE (green) and TC-core (red) colocalization at a crescent shaped core accumulation (inset), presumably at a lipid droplet. Scale bar 10 µm.
(TIF)

Figure S13 **Vamp-1 and ApoE vesicle dynamics.** Huh-7.5 cells were transfected with ApoE-GFP or GFP-VAMP1 constructs. 200ms GFP exposures were taken by confocal microscopy every 2 seconds. Single GFP puncta were tracked using the manual tracking plugin for Image J. Plotted are average (A) velocity (µm/sec) and (B) run length (µm). Overall, 16 VAMP1-GFP vesicles (59 total runs) and 15 ApoE-GFP vesicles (90 total runs) were tracked. Error bars indicate standard error of the mean.
(TIF)

Table S1 **Genes and siRNAs tested in the RNA interference screen.**
(DOC)

Table S2 **Real time RT-PCR assays for quantifying host gene RNA levels.**
(DOC)

Video S1 **Dynamics of TC-core puncta.** Snapshot of this Video is shown in Figure 2C. Playback time is 10x real speed.
(AVI)

Video S2 **TC-core dynamics.** Montage of single TC-core puncta transport shown in Figure 4A. Playback time is 10x real speed.
(AVI)

Video S3 **TC-core dynamics in bicistronic background.** Montage of single TC-core puncta transport shown in Figure 4A. Playback time is 10x real speed.
(AVI)

Video S4 **TC-core dynamics in ΔNS2 background.** Montage of single TC-core puncta transport shown in Figure 4A. Playback time is 10x real speed.
(AVI)

Video S5 **TC-core dynamics and microtubules stained with Tubulin Tracker green.** Shown as montage in Figure 7A. Playback time is 10x real speed.
(AVI)

Video S6 **TC-core dynamics in the presence of nocodazole.** Shown as a montage in Figure 7B.
(AVI)

Video S7 **TC-core traffics with ApoE-GFP.** Shown as a montage in Figure 9A. Snapshot of whole cell shown in Figure S2.
(AVI)

Video S8 **TC-core traffics with GFP-Rab11a.** Shown as a montage in Figure 9B. Snapshot of whole cell shown in Figure S3.
(AVI)

Video S9 **TC-core traffics with Alexa Fluor 488 transferrin.** Shown as a montage in Figure 9C. Snapshot of whole cell shown in Figure S4.
(AVI)

Video S10 **TC-core traffics with GFP-VAMP1.** Shown as a montage in Figure 9D. Snapshot of whole cell shown in Figure S5.
(AVI)

Video S11 **TC-core traffics with dextran.** Shown as a montage in Figure 9E. Snapshot of whole cell shown in Figure S6.
(AVI)

Video S12 **TC-core traffics with Ribogreen labeled RNA.** Shown as montage in Figure 2D.
(MOV)

Video S13 **TC-core in infected cells traffics with ApoE-GFP.** Shown as a montage in S11.
(AVI)

Video S14 **TC-core in infected cells traffics with GFP-Rab11a.** Shown as a montage in S11.
(MOV)

Video S15 **TC-core in infected cells traffics with GFP-Vamp1.** Shown as a montage in S11.
(MOV)
