# Supplementary figures and images for: Molecular Determinants and Dynamics of Hepatitis C Virus Secretion
Source: PLoS Pathog. 2012 Jan 5;8(1):e1002466. doi: 10.1371/journal.ppat.1002466 (PMC3252379; doi:10.1371/journal.ppat.1002466)

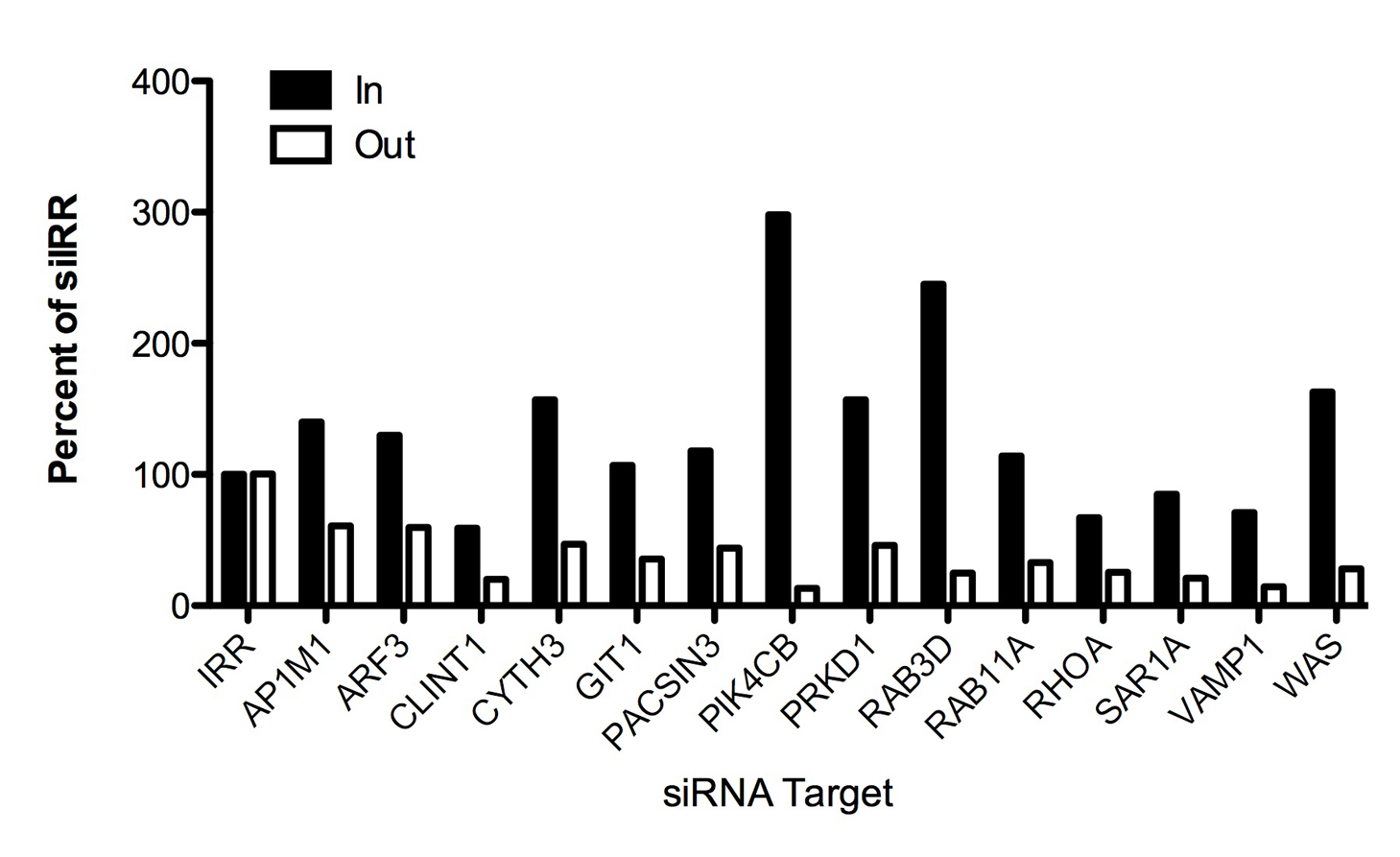

Supplement: Figure S2 — Intra- and extra-cellular infectious virus following siRNA treatment. Relative levels of intra-and extra-cellular infectious virus levels from Table 1. (TIF) [file ppat.1002466.s002.tif]

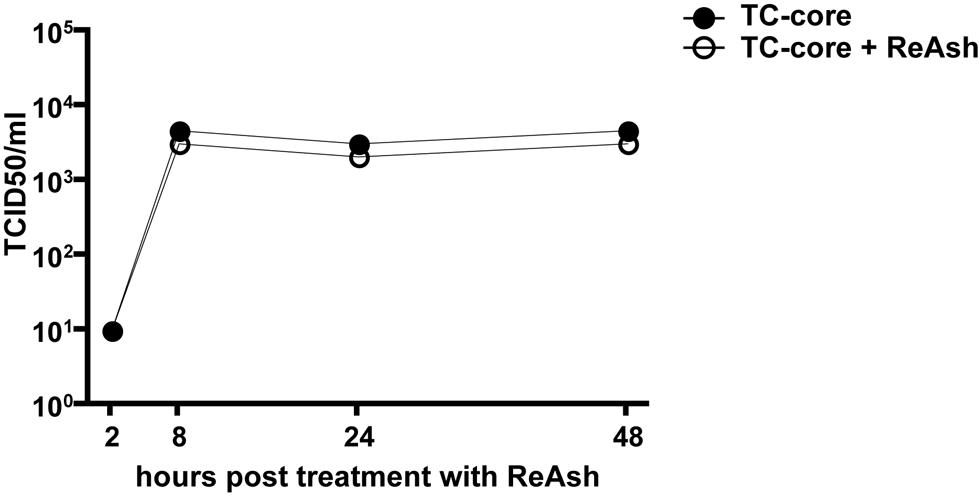

Supplement: Figure S3 — Biarsenical dyes do not affect virus release. Huh-7.5 cells were infected with wildtype virus for 48 hours then incubated with ReAsh dye for 30 minutes. ReAsh dye was removed and cells were washed with 1x BAL buffer supplemented with 500 µM EDT. Cells were incubated in fresh media and supernatants were collect at 2, 8, 24, 48 hours post ReAsh incubation and titered. (TIF) [file ppat.1002466.s003.tif]

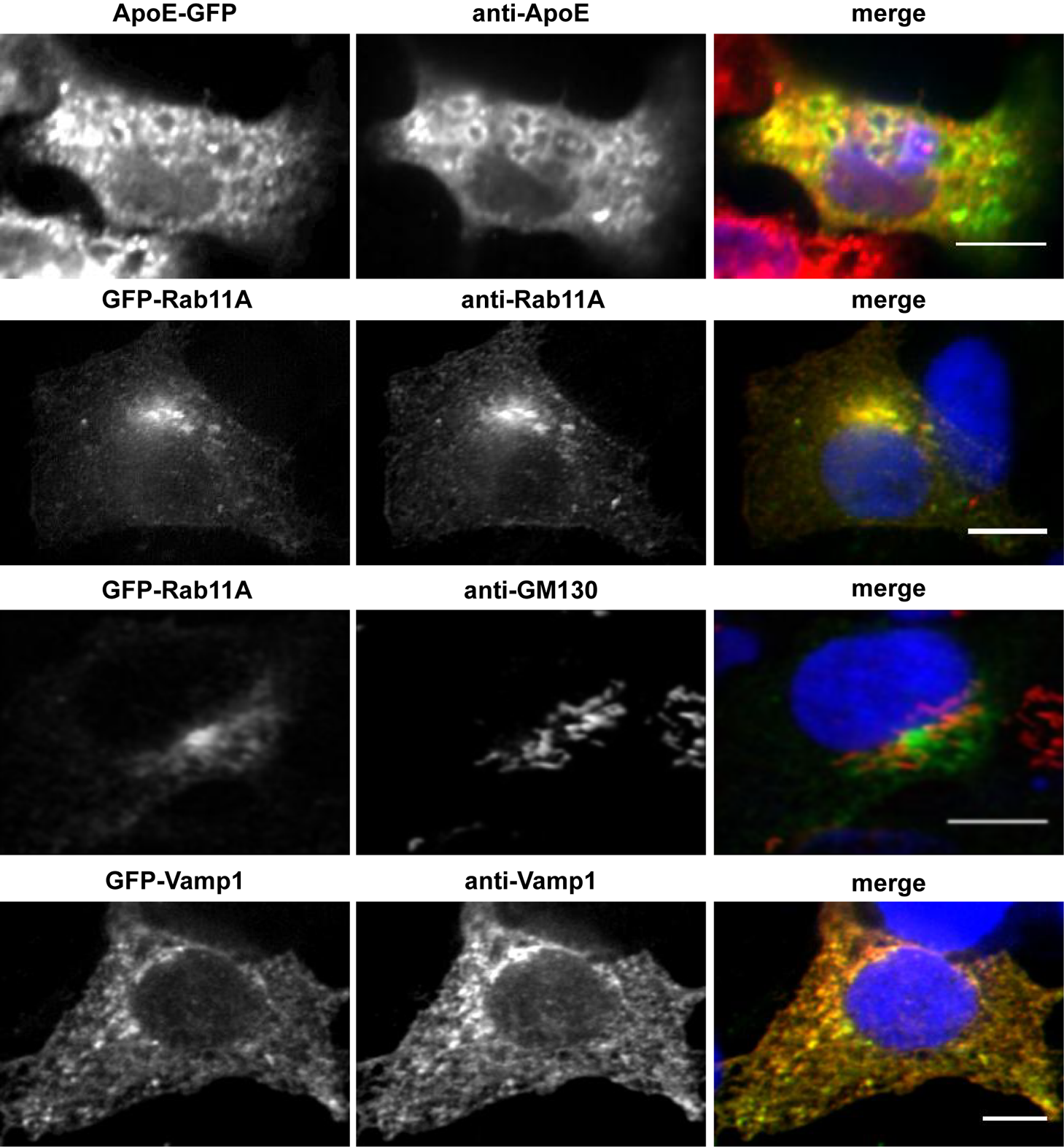

Supplement: Figure S4 — Immunofluorescence of GFP fusions of ApoE, Vamp1, and Rab11a. Huh7.5 cells were transfected with ApoE-GFP, GFP-Vamp1, or GFP-Rab11a. Cells were fixed and processed for immunofluorescence using antibodies directed against ApoE (top), Rab11a (middle), GM130 (middle bottom) or Vamp1 (bottom) and corresponding secondary antibodies (red). Scale bar is 10 µm. (TIF) [file ppat.1002466.s004.tif]

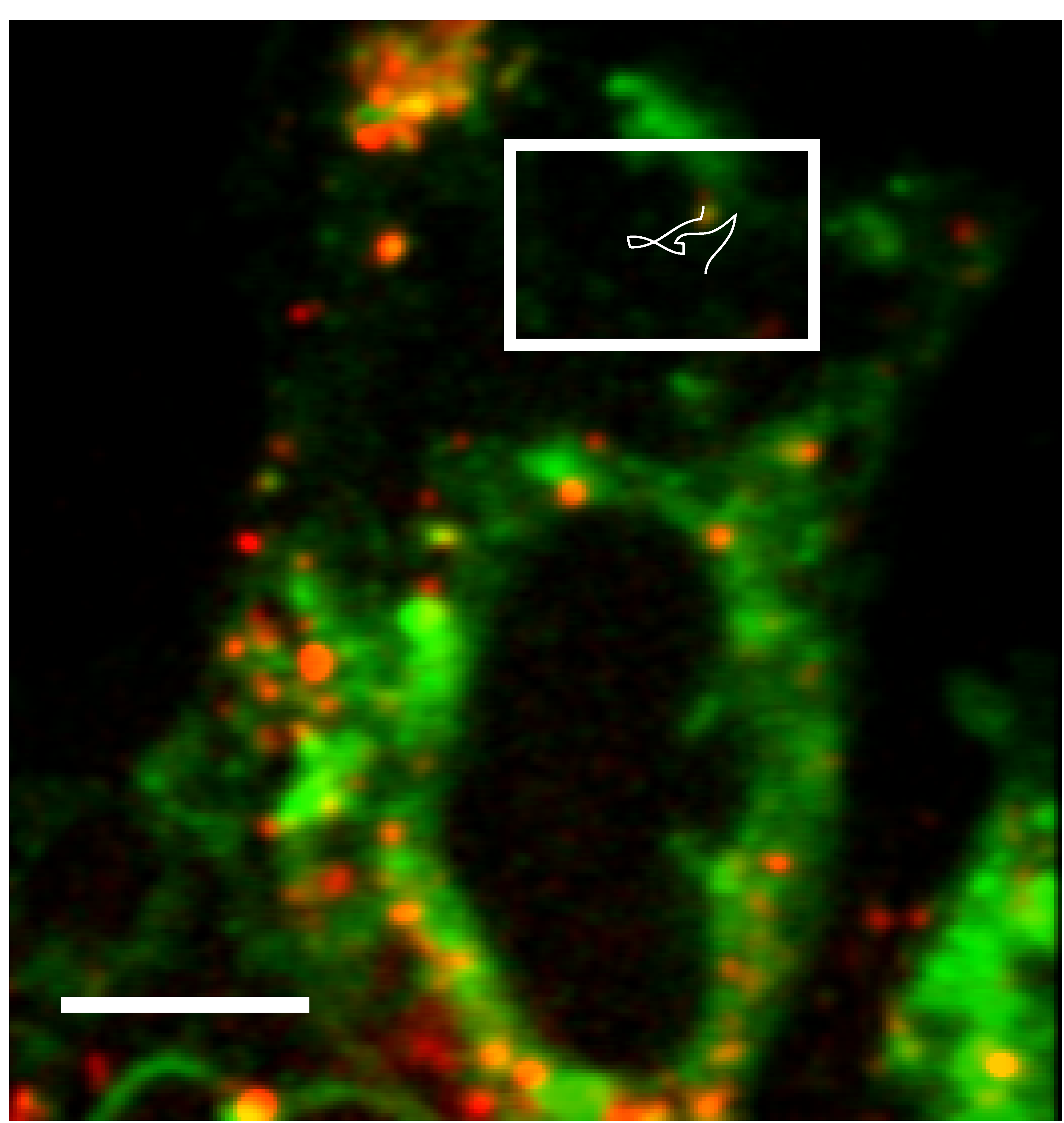

Supplement: Figure S5 — TC-core cotransports with ApoE-GFP. Huh-7.5 cells were electroporated with TC-core RNA and transfected with ApoE-GFP at 48 hpe. Cells were stained with ReAsh (red) at 72 hpe then imaged. Boxed region contains TC-core puncta shown in montage in Figure 9A and as a Video S7. Line indicates trajectory of moving particle. Scale bar = 10 µm. (TIF) [file ppat.1002466.s005.tif]

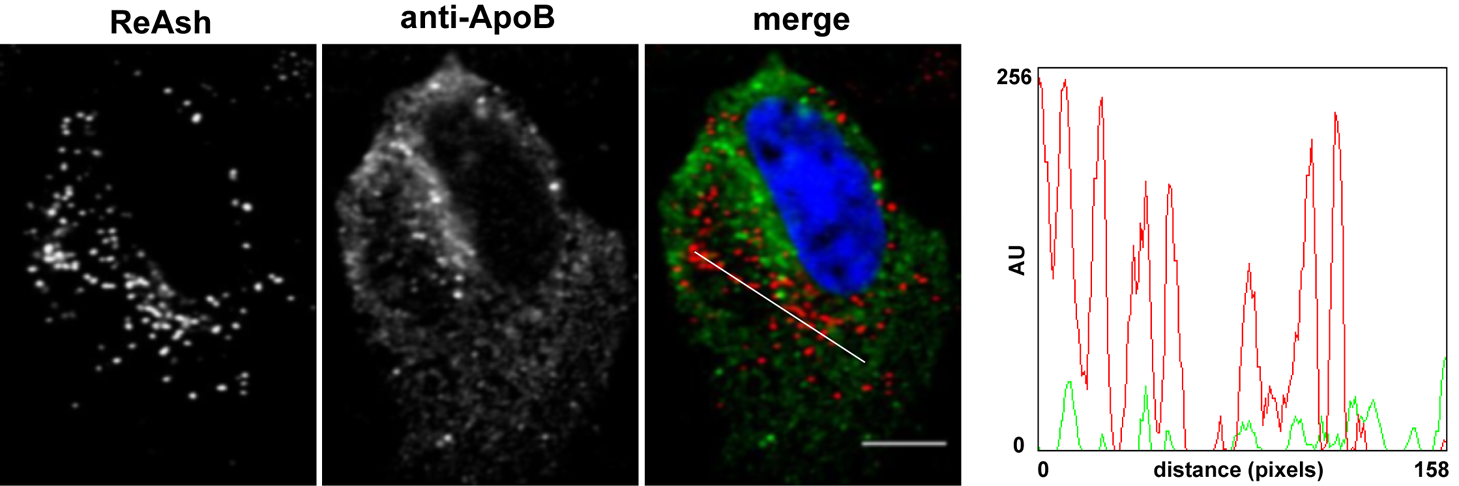

Supplement: Figure S6 — ApoB does not colocalize with TC-core puncta. Huh7.5 cells were infected with TC-core virus and stained with ReAsh dye (red) at 72 hours post infection followed by processing for immunofluorescence using an ApoB antibody (green). Scale bar is 10 µm. (TIF) [file ppat.1002466.s006.tif]

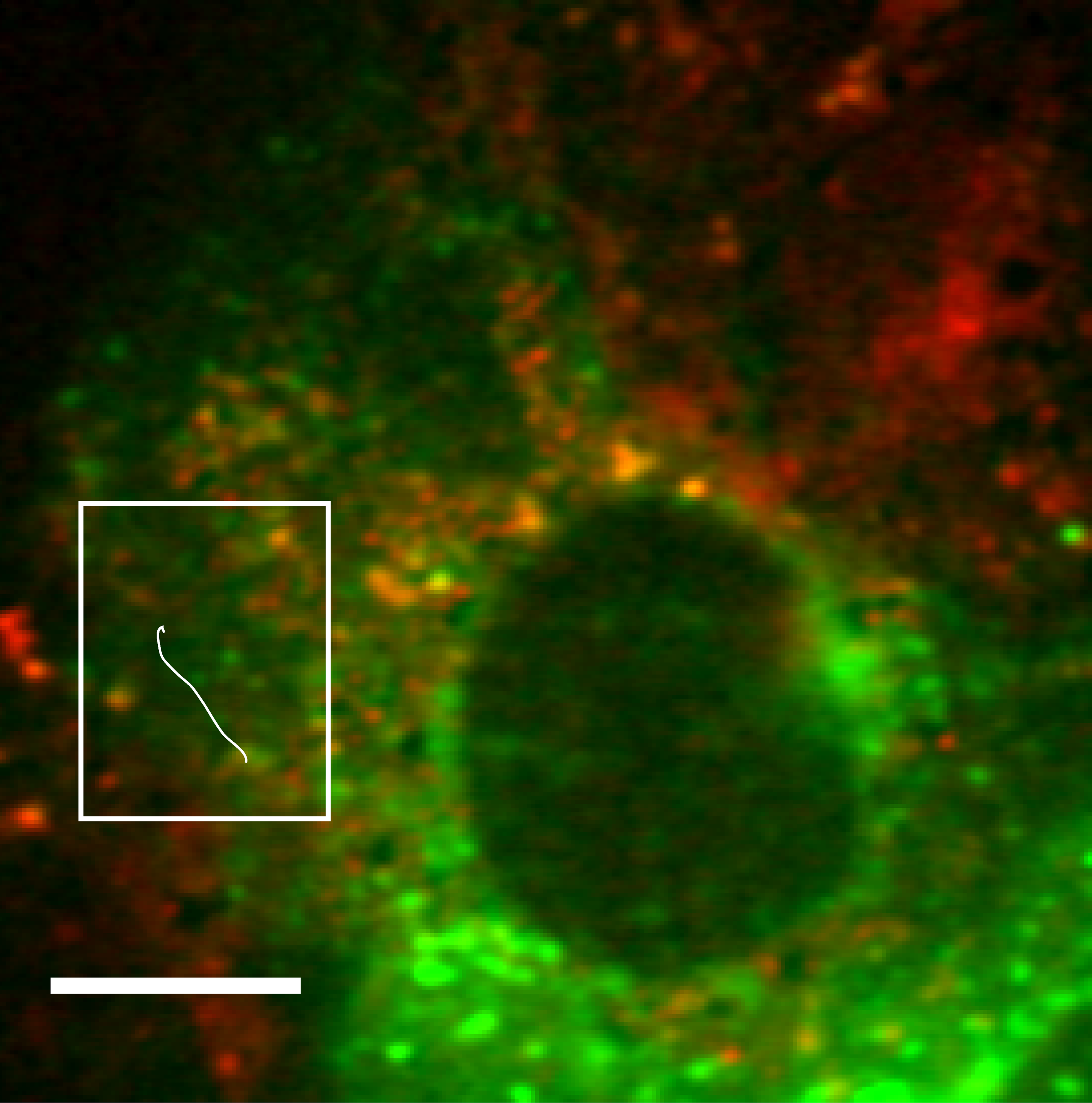

Supplement: Figure S7 — TC-core cotransports with GFP-Rab11a. Huh-7.5 cells were electroporated with TC-core RNA and transfected with GFP-Rab11a at 48 hpe. Cells were stained with ReAsh (red) at 72 hpe then imaged. Boxed region contains TC-core puncta shown in montage in Figure 9B and as a Video S8. Line indicates trajectory of moving particle. Scale bar = 10 µm. (TIF) [file ppat.1002466.s007.tif]

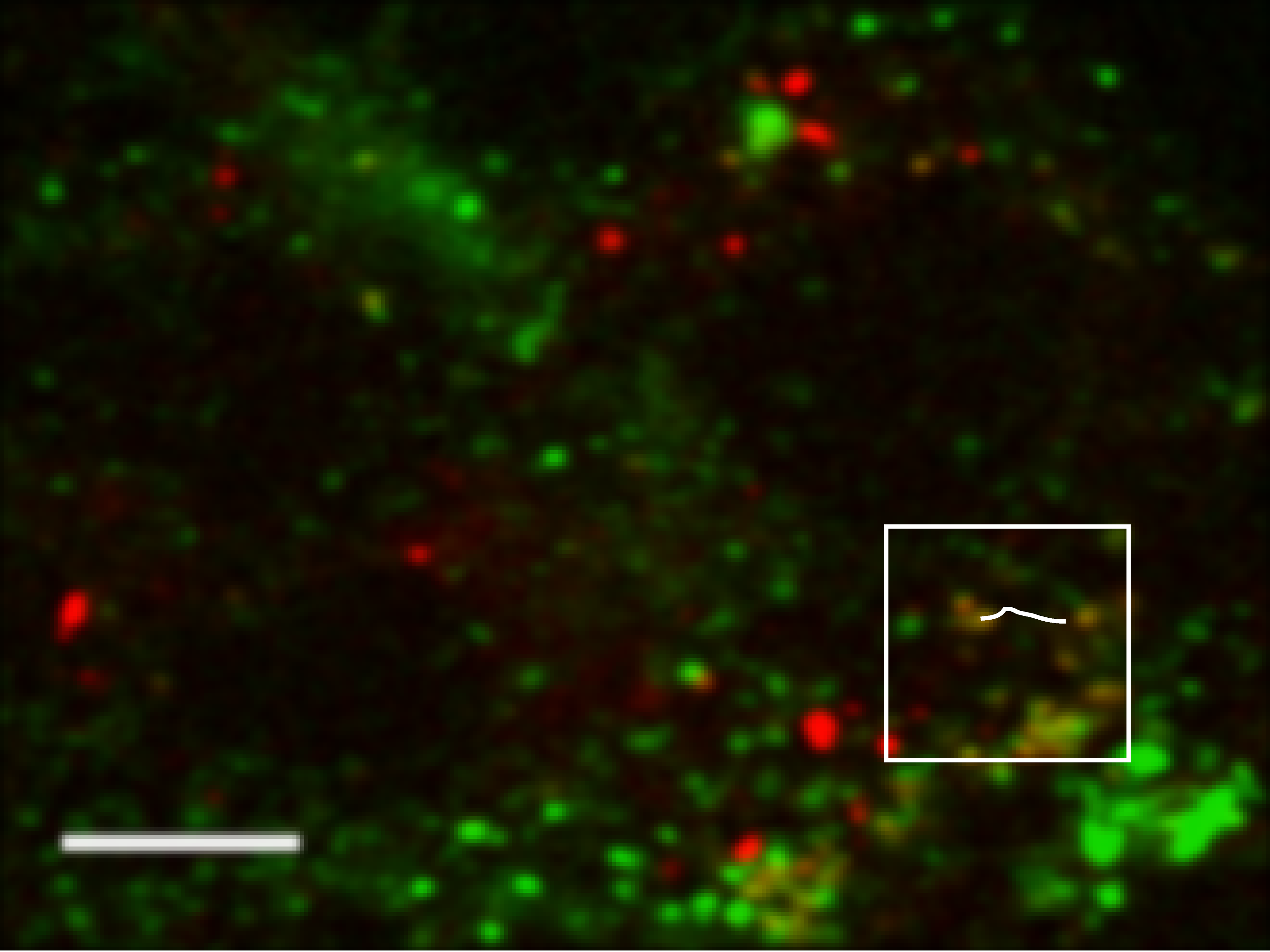

Supplement: Figure S8 — TC-core cotransports with Alexa Fluor 488 transferrin. Huh-7.5 cells were electroporated with TC-core RNA and stained with ReAsh (red) at 72 hpe then incubated with Alexa Fluor 488 transferrin (green). Cells were immediately imaged. Boxed region contains TC-core puncta shown in montage in Figure 9C and as a Video S9. Line indicates trajectory of moving particle. Scale bar = 10 µm. (TIF) [file ppat.1002466.s008.tif]

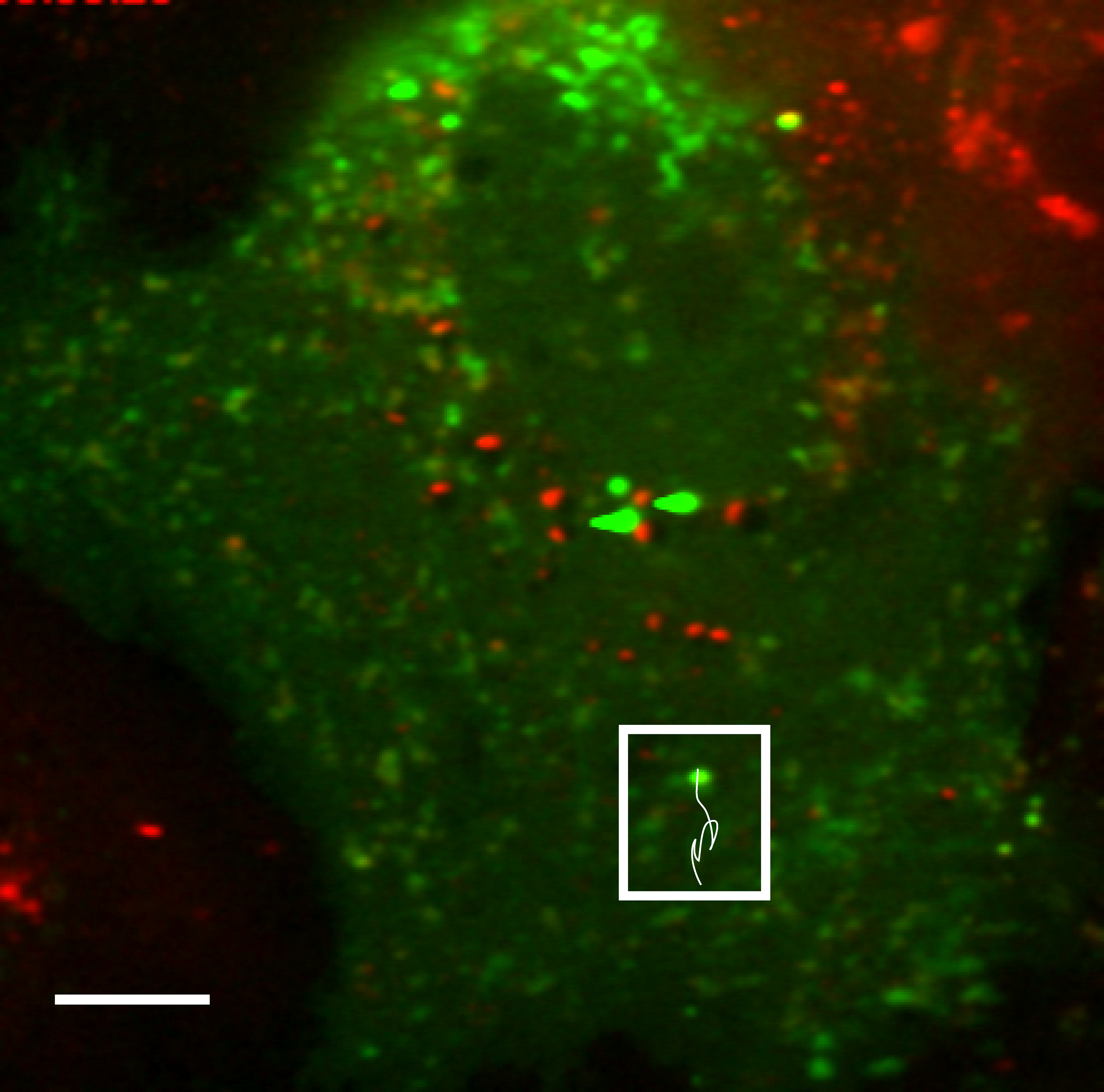

Supplement: Figure S9 — TC-core cotransports with GFP-VAMP1. Huh-7.5 cells were electroporated with TC-core RNA and transfected with GFP-VAMP1 at 48 hpe. Cells were stained with ReAsh (red) at 72 hpe then imaged. Boxed region contains TC-core puncta shown in montage in Figure 9D and as a Video S10. Line indicates trajectory of moving particle. Scale bar = 10 µm. (TIF) [file ppat.1002466.s009.tif]

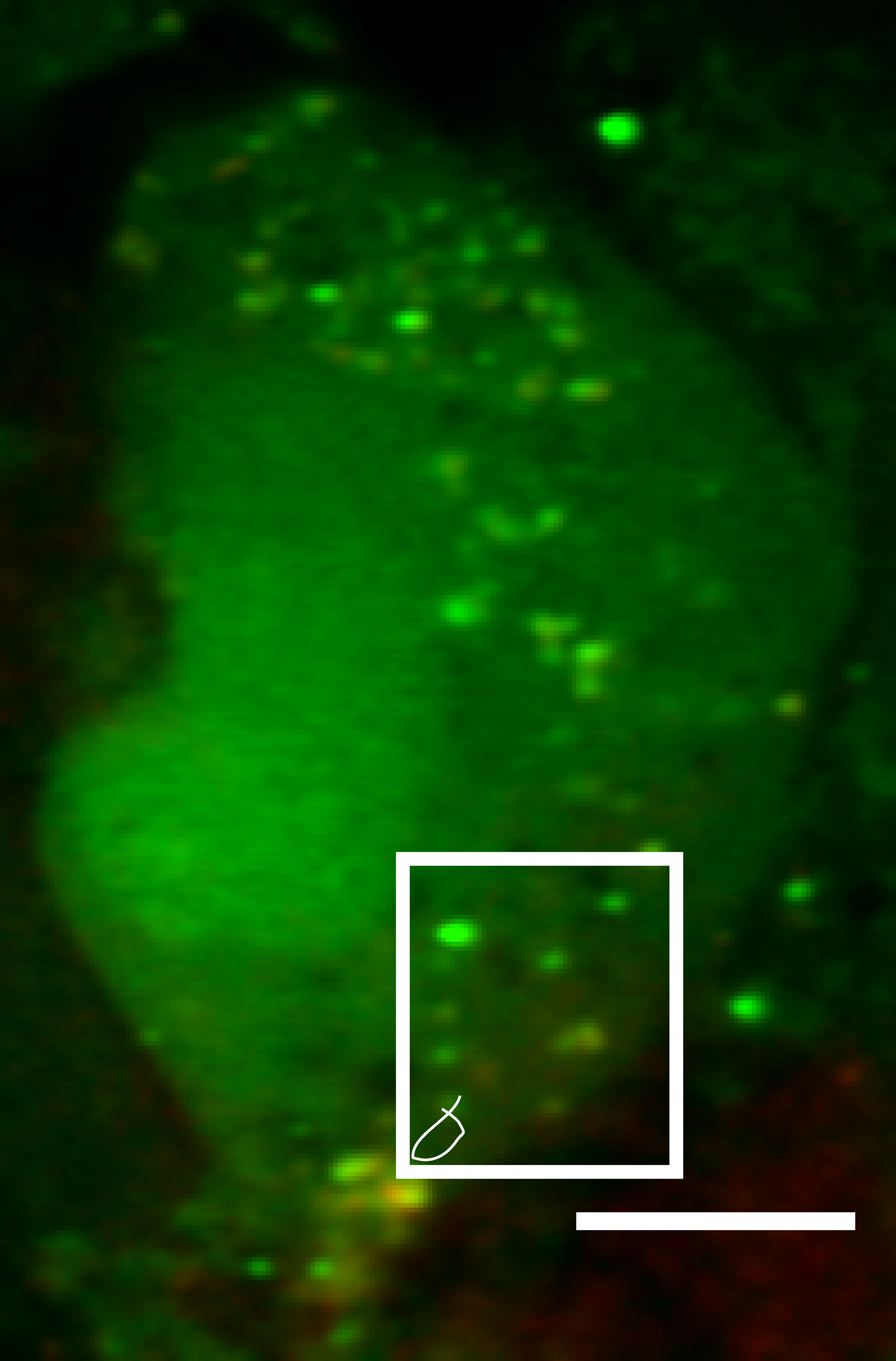

Supplement: Figure S10 — TC-core cotransports with dextran. Huh-7.5 cells were electroporated with TC-core RNA and stained with FlAsh (green) at 72 hpe then incubated with dextran (red). Cells were immediately imaged. Boxed region contains TC-core puncta shown in montage in Figure 9E and as a Video S11. Line indicates trajectory of moving particle. Scale bar = 10 µm. (TIF) [file ppat.1002466.s010.tif]

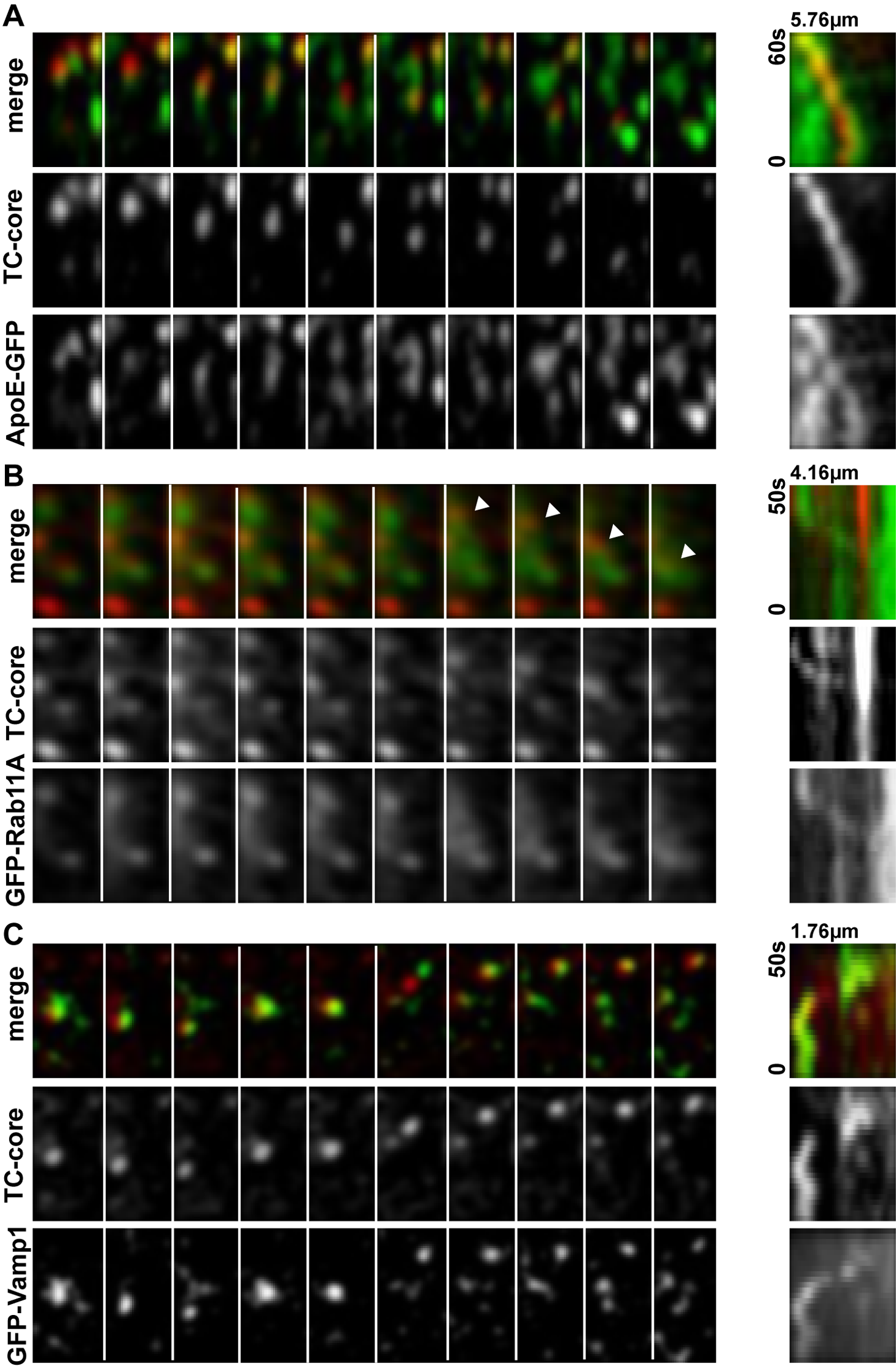

Supplement: Figure S11 — TC-core produced in infected cells co-transports with host secretory pathway components. Huh-7.5 cells were infected with TC-core virus followed by transfection with either ApoE-GFP (A), GFP-Rab11a (B), or GFP-Vamp1 (C) plasmids at 24 hours post infection (hpi). Cells were incubated with ReAsh at 72hpi followed by live cell confocal microscopy. Shown are time-lapse montages of alternating DsRed (200ms) and EGFP (200ms) exposures taken every 2 seconds for several minutes. Kymographs are shown to the right of each montage. (TIF) [file ppat.1002466.s011.tif]

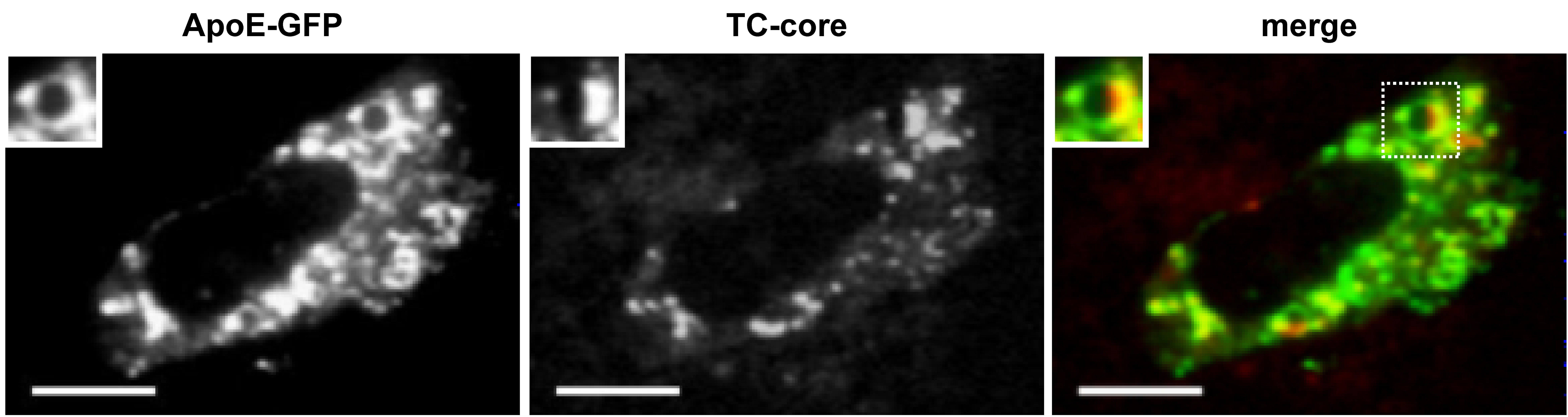

Supplement: Figure S12 — ApoE-TC-core colocalization at core accumulations. Huh-7.5 cells were electroporated with TC-core RNA and transfected with ApoE-GFP at 48 hours post electroporation. Shown is ApoE (green) and TC-core (red) colocalization at a crescent shaped core accumulation (inset), presumably at a lipid droplet. Scale bar 10 µm. (TIF) [file ppat.1002466.s012.tif]

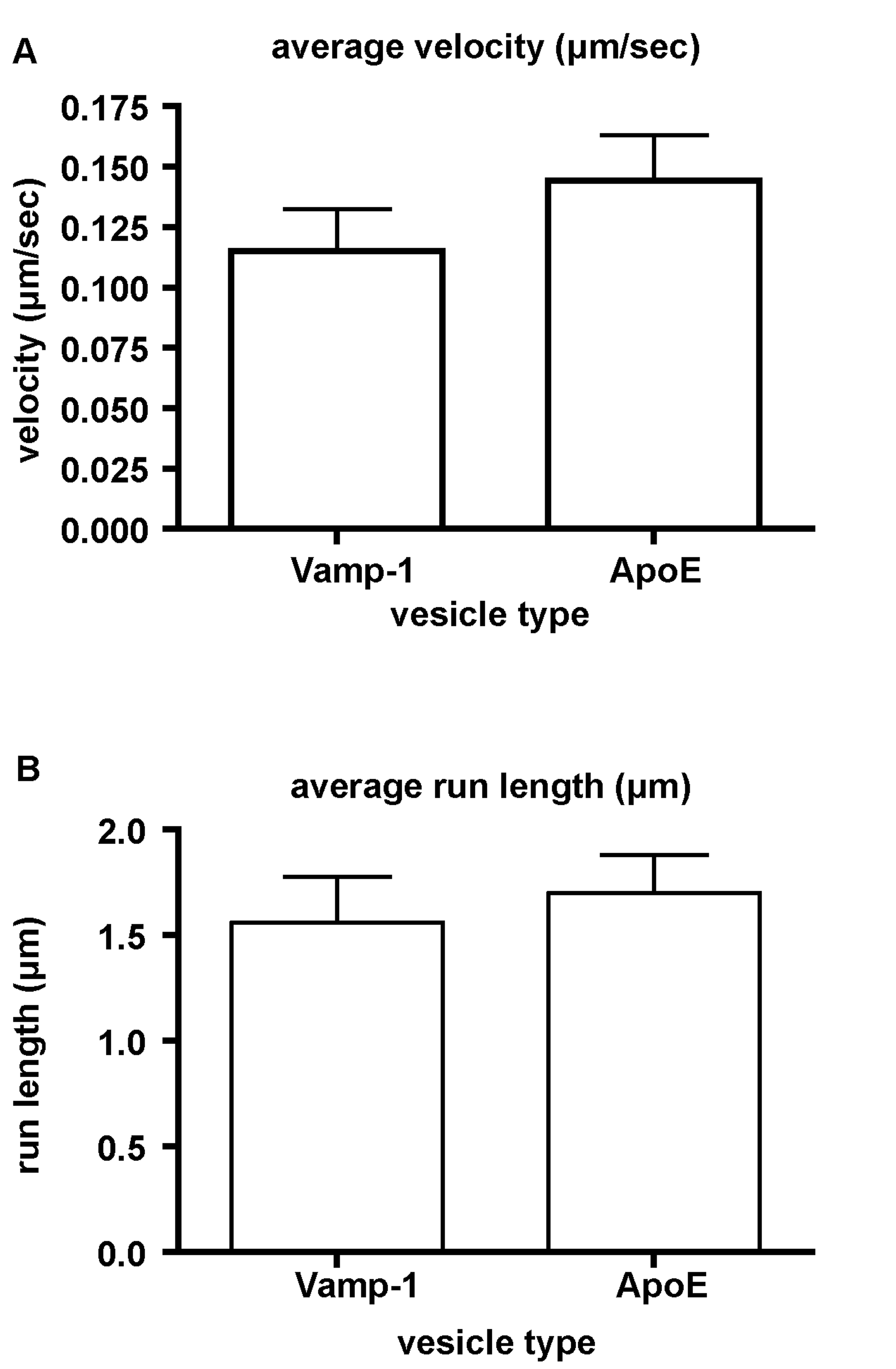

Supplement: Figure S13 — Vamp-1 and ApoE vesicle dynamics. Huh-7.5 cells were transfected with ApoE-GFP or GFP-VAMP1 constructs. 200ms GFP exposures were taken by confocal microscopy every 2 seconds. Single GFP puncta were tracked using the manual tracking plugin for Image J. Plotted are average (A) velocity (µm/sec) and (B) run length (µm). Overall, 16 VAMP1-GFP vesicles (59 total runs) and 15 ApoE-GFP vesicles (90 total runs) were tracked. Error bars indicate standard error of the mean. (TIF) [file ppat.1002466.s013.tif]
