# Supplementary material for: Molecular Determinants and Dynamics of Hepatitis C Virus Secretion
Source: PLoS Pathog. 2012 Jan 5;8(1):e1002466. doi: 10.1371/journal.ppat.1002466 (PMC3252379; doi:10.1371/journal.ppat.1002466)
Supplement: Table S1 — Genes and siRNAs tested in the RNA interference screen. (DOC) [file ppat.1002466.s014.doc]

**SI Table 1.** Genes and siRNAs tested in RNA interference screen.

| **Gene** | **GenBank**  **Accession No.** | **siGENOME SMARTpool**  **(Primary Screen)** | **siGENOME UPGRADE** | | |
| --- | --- | --- | --- | --- | --- |
|  |  | **Pooled siRNAs** | **Catalog No.** | | **Individual siRNAs** |
| SAR1A | NM_020150 | GAGCAAGCACGUCGCGUUU | D-016756-01^a^ | | GAGCAAGCACGUCGCGUUU |
|  |  | UUAAUGGGAUUGUCUUUCU | D-016756-02 | | UUAAUGGGAUUGUCUUUCU ^b^ |
|  |  | CAAUAUCCAAUGUGCCAAU | D-016756-04* | | CUGGAAAACUUGUAUUCUU ^b^ |
|  |  | CUGGAAAACUUGUAUUCUU | D-016756-17* | | GUGCAUGCAUUUCGUUUAU ^b,c^ |
| CYTH3 | NM_004227 | GGAGAAGGCCUAAAUAAGA | D-019268-01 | | GGAGAAGGCCUAAAUAAGA ^b^ |
|  |  | CAGCAGAGAUCCCUUCUAU | D-019268-02 | | CAGCAGAGAUCCCUUCUAU |
|  |  | GAAGACCUCUCAUUAGAAG | D-019268-03 | | GAAGACCUCUCAUUAGAAG |
|  |  | GGAAUCAUCCCGUUGGAAA | D-019268-04 | | GGAAUCAUCCCGUUGGAAA ^b,c^ |
| CLINT1 | NM_014666 | GAUCACAGAAUACAGAUAU | D-021406-01 | | GAUCACAGAAUACAGAUAU ^b,c^ |
|  |  | GAUCAGAGCGUGUUGUUAC | D-021406-02 | | GAUCAGAGCGUGUUGUUAC ^b,c^ |
|  |  | UACGAUCCCUGGAAAAUUA | D-021406-03 | | UACGAUCCCUGGAAAAUUA ^b,c^ |
|  |  | GGGAUGAGGAGUGGGAUAA | D-021406-04 | | GGGAUGAGGAGUGGGAUAA ^b,c^ |
| PRKD1 | NM_002742 | GAAGAGAUGUAGCUAUUAA | D-005028-01 | | GAAGAGAUGUAGCUAUUAA |
|  |  | GAAAGAGUGUUUGUUGUUA | D-005028-02 | | GAAAGAGUGUUUGUUGUUA ^b,c^ |
|  |  | GAAUGCAGCUUUCAUGUAU | D-005028-03 | | GAAUGCAGCUUUCAUGUAU |
|  |  | GGAAGGAAAUAUCUCAUGA | D-005028-21* | | UCGAAAUCACUACGGCAAA ^b^ |
| PI4KB | NM_002651 | GGACGUGGGUGAUGCCAUU | D-006777-01 | | GGACGUGGGUGAUGCCAUU ^b,c^ |
|  |  | GGGAUGACCUUCGGCAAGA | D-006777-02 | | GGGAUGACCUUCGGCAAGA ^b,c^ |
|  |  | GAGAUCCGUUGCCUAGAUG | D-006777-03 | | GAGAUCCGUUGCCUAGAUG ^b,c^ |
|  |  | GCACCGAGAGUAUUGAUAA | D-006777-06 | | GCACCGAGAGUAUUGAUAA ^b,c^ |
| AP1M1 | NM_032493 | CAACGACAAGGUCCUCUUU | D-013196-01 | | CAACGACAAGGUCCUCUUU |
|  |  | GAGAUCGUGUGGUCCAUCA | D-013196-02 | | GAGAUCGUGUGGUCCAUCA ^b,c^ |
|  |  | UAUCACGCUUCGAGAAUGA | D-013196-03 | | UAUCACGCUUCGAGAAUGA ^b^ |
|  |  | UCACGCAGAAUGGAGAUUA | D-013196-18* | | GCCCAAUGAUGCCGACUCA |
| VAMP1 | NM_014231 | CUCCUAACAUGACCAGUAA | D-012497-01 | | CUCCUAACAUGACCAGUAA ^b,c^ |
|  |  | CAUCACAAUUUGAGAGCAG | D-012497-02 | | CAUCACAAUUUGAGAGCAG ^b,c^ |
|  |  | CCAUCAUCGUGGUAGUUAU | D-012497-03 | | CCAUCAUCGUGGUAGUUAU ^b,c^ |
|  |  | AGGCACAAGUGGAGGAGGU | D-012497-04 | | AGGCACAAGUGGAGGAGGU ^b,c^ |
| RAB11A | NM_004663 | GUAGGUGCCUUAUUGGUUU | D-004726-01 | | GUAGGUGCCUUAUUGGUUU ^b^ |
|  |  | GCAACAAUGUGGUUCCUAU | D-004726-02 | | GCAACAAUGUGGUUCCUAU ^b,c^ |
|  |  | CAAGAGCGAUAUCGAGCUA | D-004726-03 | | CAAGAGCGAUAUCGAGCUA |
|  |  | GGAGUAGAGUUUGCAACAA | D-004726-06 | | GGAGUAGAGUUUGCAACAA |
| RAB3D | NM_004283 | UGACAUCGCCAAUCAGGAA | D-010822-03 | | GUUCAAACUGCUACUGAUA ^b^ |
|  |  | GUUCAAACUGCUACUGAUA | D-010822-04 | | GUACUGUGGGCAUCGAUUU ^b^ |
|  |  | GUACUGUGGGCAUCGAUUU | D-010822-05 | | GGACGAACGUGUUGUGCCU ^b,c^ |
|  |  | GGACGAACGUGUUGUGCCU | D-010822-18* | | CGGCAGGGACUGUGAUCGA ^b^ |
| WAS | NM_000377 | CGAGAACCAGCGACUCUUU | D-028294-05 | | CGAGAACCAGCGACUCUUU ^b,c^ |
|  |  | GCUGGCCACUGCAGUUGUU | D-028294-06 | | GCUGGCCACUGCAGUUGUU ^b,c^ |
|  |  | ACUCACAGCUUGUCUACUC | D-028294-08* | | GAACAUACCCUCCACCCUC ^b,c^ |
|  |  | GAACAUACCCUCCACCCUC | D-028294-21* | | CCUCUAAACUUAUCUACGA ^b,c^ |
| PACSIN3 | NM_016223 | GGACAUGGAACAGGCCUUU | D-015343-01 | | GGACAUGGAACAGGCCUUU |
|  |  | CCAACUACGUGGAGUGUGU | D-015343-02 | | CCAACUACGUGGAGUGUGU ^b,c^ |
|  |  | ACAAUCAGCCGGAAAGAGA | D-015343-03 | | ACAAUCAGCCGGAAAGAGA |
|  |  | GGAUAUGCUGCUCACCUUA | D-015343-18* | | UGAGGCAGCCAGUGACGAA ^b^ |
| ARF3 | NM_001659 | GGGAAGAGCUGAUGAGAAU | D-011581-01 | | GGGAAGAGCUGAUGAGAAU |
|  |  | GGAAAGACCACCAUCCUAU | D-011581-02 | | GGAAAGACCACCAUCCUAU ^b^ |
|  |  | UAUGAACGCUGCUGAGAUC | D-011581-03 | | UAUGAACGCUGCUGAGAUC ^b,c^ |
|  |  | GCAAUGAUCGGGAGCGAGU | D-011581-04 | | GCAAUGAUCGGGAGCGAGU ^b^ |
| RHOA | NM_001664 | AUGGAAAGCAGGUAGAGUU | D-003860-01 | | AUGGAAAGCAGGUAGAGUU |
|  |  | GAACUAUGUGGCAGAUAUC | D-003860-02 | | GAACUAUGUGGCAGAUAUC |
|  |  | GAAAGACAUGCUUGCUCAU | D-003860-03 | | GAAAGACAUGCUUGCUCAU ^b^ |
|  |  | GAGAUAUGGCAAACAGGAU | D-003860-04 | | GAGAUAUGGCAAACAGGAU ^b,c^ |
| GIT1 | NM_014030 | GGACGACGCCAUCUAUUCA | D-020565-02 | | GGACGACGCCAUCUAUUCA |
|  |  | GCACACCCAUUGACUAUGC | D-020565-03 | | GCACACCCAUUGACUAUGC ^b^ |
|  |  | GGACGCCACAUCUCCAUUG | D-020565-04 | | GGACGCCACAUCUCCAUUG |
|  |  | CCGCACACCCAUUGACUAU | D-020565-05 | | CCGCACACCCAUUGACUAU ^b,c^ |
| ACACA | NM_198834 | M-004551-02 ^a^ |  | |  |
| ACTR2 | NM_005722 | M-012076-00 |  | |  |
| ACTR3 | NM_005721 | M-012077-01 |  | |  |
| ADAM10 | NM_001110 | M-004503-01 |  | |  |
| AMPH | NM_001635 | M-011569-00 |  | |  |
| AP1B1 | NM_001127 | M-011200-00 |  | |  |
| AP1M2 | NM_005498 | M-012056-00 |  | |  |
| AP2A1 | NM_014203 | M-012492-00 |  | |  |
| AP2A2 | NM_012305 | M-012812-00 |  | |  |
| AP2B1 | NM_001282 | M-003627-01 |  | |  |
| AP2M1 | NM_004068 | M-008170-00 |  | |  |
| ARF1 | NM_001658 | M-011580-00 |  | |  |
| ARF5 | NM_001662 | M-011584-00 |  | |  |
| ARF6 | NM_001663 | M-004008-00 |  | |  |
| ARFIP2 | NM_012402 | M-012820-00 |  | |  |
| ARPC1B | NM_005720 | M-012082-01 |  | |  |
| ARPC2 | NM_005731 | M-012081-00 |  | |  |
| ARPC3 | NM_005719 | M-005284-00 |  | |  |
| ARPC4 | NM_005718 | M-008571-00 |  | |  |
| ARPC5 | NM_005717 | M-012080-00 |  | |  |
| ARRB1 | NM_004041 | M-011971-00 |  | |  |
| ARRB2 | NM_004313 | M-007292-00 |  | |  |
| ATG5 | NM_004849 | M-004374-03 |  | |  |
| ATG12 | NM_004707 | M-010212-02 |  | |  |
| ATM | NM_138293 | M-003201-04 |  | |  |
| ATP6V0A1 | NM_005177 | M-017618-00 |  | |  |
| BIN1 | NM_004305 | M-008246-00 |  | |  |
| CAMK1 | NM_003656 | M-004940-00 |  | |  |
| CAV1 | NM_001753 | M-003467-01 |  | |  |
| CAV2 | NM_001233 | M-010958-00 |  | |  |
| CAV3 | NM_001234 | M-011229-00 |  | |  |
| CBL | NM_005188 | M-003003-01 |  | |  |
| CBLB | NM_170662 | M-003004-01 |  | |  |
| CBLC | NM_012116 | M-006962-00 |  | |  |
| CDC42 | NM_001791 | M-005057-00 |  | |  |
| CFL1 | NM_005507 | M-012707-00 |  | |  |
| CIB1 | NM_006384 | M-012261-00 |  | |  |
| CIB2 | NM_006383 | M-012230-00 |  | |  |
| CIB3 | NM_054113 | M-012901-00 |  | |  |
| CLTA | NM_001833 | M-004002-00 |  | |  |
| CLTB | NM_001834 | M-004003-00 |  | |  |
| CLTC | NM_004859 | M-004001-00 |  | |  |
| CLTCL1 | NM_001835 | M-011611-00 |  | |  |
| COPA | NM_004371 | M-011835-00 |  | |  |
| COPB2 | NM_004766 | M-019847-01 |  | |  |
| CTBP1 | NM_001328 | M-008609-01 |  | |  |
| DAB2 | NM_001343 | M-008522-00 |  | |  |
| DDEF2 | NM_003887 | M-011544-00 |  | |  |
| DIAPH1 | NM_005219 | M-010347-02 |  | |  |
| DNM1 | NM_004408 | M-003940-00 |  | |  |
| DNM2 | NM_004945 | M-004007-01 |  | |  |
| DNM3 | NM_015569 | M-013931-00 |  | |  |
| EEA1 | NM_003566 | M-004012-01 |  | |  |
| EFS | NM_005864 | M-012094-00 |  | |  |
| ELKS | NM_015064 | M-010942-00 |  | |  |
| EPN1 | NM_013333 | M-004724-00 |  | |  |
| EPN2 | NM_148921 | M-004725-00 |  | |  |
| EPN3 | NM_017957 | M-021006-00 |  | |  |
| EPS15 | NM_001981 | M-004005-00 |  | |  |
| EPS15L1 | NM_021235 | M-004006-00 |  | |  |
| FASN | NM_004104 | M-003954-04 |  | |  |
| FYN | NM_002037 | M-003140-03 |  | |  |
| GAF1 | NM_015470 | M-004298-00 |  | |  |
| GNB2L1 | NM_006098 | M-006876-00 |  | |  |
| GORASP1 | NM_031899 | M-013510-00 |  | |  |
| GRB2 | NM_002086 | M-019220-00 |  | |  |
| HGS | NM_004712 | M-016835-00 |  | |  |
| HIP1 | NM_005338 | M-005001-01 |  | |  |
| HIP1R | NM_003959 | M-027079-00 |  | |  |
| IHPK3 | NM_054111 | M-006739-00 |  | |  |
| ITSN1 | NM_003024 | M-008365-00 |  | |  |
| ITSN2 | NM_006277 | M-009841-00 |  | |  |
| LIMK1 | NM_002314 | M-007730-02 |  | |  |
| MAP4K2 | NM_004579 | M-003587-01 |  | |  |
| MAPK8IP1 | NM_005456 | M-003595-00 |  | |  |
| MAPK8IP2 | NM_012324 | M-012462-00 |  | |  |
| MAPK8IP3 | NM_015133 | M-003596-01 |  | |  |
| NEDD4 | NM_006154 | M-007178-01 |  | |  |
| NEDD4L | NM_015277 | M-007187-01 |  | |  |
| NSF | NM_006178 | M-009401-00 |  | |  |
| PACSIN1 | NM_020804 | M-007735-00 |  | |  |
| PAK1 | NM_002576 | M-003521-03 |  | |  |
| PI4KII | NM_018425 | M-006770-01 |  | |  |
| PI4K2B | NM_018323 | M-006769-01 |  | |  |
| PICALM | NM_007166 | M-004004-02 |  | |  |
| PIK3C2G | NM_004570 | M-006773-00 |  | |  |
| PIK3CG | NM_002649 | M-005274-02 |  | |  |
| PIK4CA | NM_002650 | M-006776-03 |  | |  |
| PIP5K1A | NM_003557 | M-004780-02 |  | |  |
| PITPNM1 | NM_004910 | M-019888-00 |  | |  |
| RAB11B | NM_004218 | M-004727-01 |  | |  |
| RAB3A | NM_002866 | M-009668-01 |  | |  |
| RAB3B | NM_002867 | M-008825-00 |  | |  |
| RAB3C | NM_138453 | M-008520-00 |  | |  |
| RAB4A | NM_004578 | M-008539-01 |  | |  |
| RAB4B | NM_016154 | M-008780-01 |  | |  |
| RAB5A | NM_004162 | M-004009-00 |  | |  |
| RAB5B | NM_002868 | M-004010-01 |  | |  |
| RAB5C | NM_004583 | M-004011-01 |  | |  |
| RAB6A | NM_002869 | M-008975-01 |  | |  |
| RAB6B | NM_016577 | M-008548-00 |  | |  |
| RAB7B | NM_177403 | M-018225-00 |  | |  |
| RAB7L1 | NM_003929 | M-010556-01 |  | |  |
| RAB8A | NM_005370 | M-003905-00 |  | |  |
| RAB8B | NM_016530 | M-008744-01 |  | |  |
| RAC1 | NM_018890 | M-003560-02 |  | |  |
| ROCK1 | NM_005406 | M-003536-01 |  | |  |
| ROCK2 | NM_004850 | M-004610-01 |  | |  |
| SH3GLB1 | NM_016009 | M-017086-00 |  | |  |
| SH3GLB2 | NM_020145 | M-015810-00 |  | |  |
| SNAP91 | NM_014841 | M-032296-00 |  | |  |
| SREBF1 | NM_004176 | M-006891-00 |  | |  |
| STAU | NM_004602 | M-011894-00 |  | |  |
| SYNJ1 | NM_003895 | M-019486-01 |  | |  |
| SYNJ2 | NM_003898 | M-012624-01 |  | |  |
| SYT1 | NM_005639 | M-020044-00 |  | |  |
| SYT2 | NM_177402 | M-018809-00 |  | |  |
| TNIK | XM_039796 | M-004542-02 |  | |  |
| VAMP2 | NM_014232 | M-012498-00 |  | |  |
| VAPA | NM_003574 | M-021382-00 |  | |  |
| VAPB | NM_004738 | M-017795-00 |  | |  |
| VAV2 | NM_003371 | M-005199-00 |  | |  |
| VIL2 | NM_003379 | M-017370-01 |  | |  |
| WASF1 | NM_003931 | M-011557-00 |  | |  |
| WASF2 | NM_006990 | M-012141-00 |  | |  |
| WASF3 | NM_006646 | M-012301-01 |  | |  |
| ^a^ Dharmacon product number  ^b^ Individual siRNA that significantly inhibited HCV  ^c^ siRNA(s) used in the In vs. Out assay | | | |  | |
| * siRNA not part of original SMARTpool for primary screen | | | |  | |
