# Supplementary material for: Molecular Determinants and Dynamics of Hepatitis C Virus Secretion
Source: PLoS Pathog. 2012 Jan 5;8(1):e1002466. doi: 10.1371/journal.ppat.1002466 (PMC3252379; doi:10.1371/journal.ppat.1002466)
Supplement: Table S2 — Real time RT-PCR assays for quantifying host gene RNA levels. (DOC) [file ppat.1002466.s015.doc]

**SI Table 2.** Real time RT-PCR assays

| Target | PCR Primer-Probe Sets^a^ |
| --- | --- |
| 18S | 4319413E |
| AP1M1 | Hs00261227_m1 |
| ARF3 | Hs00155838_m1 |
| CLINT1 | Hs00206388_m1 |
| CYTH3 | Hs00188456_m1 |
| GAPDH | 4326317E |
| GIT1 | Hs00204031_m1 |
| PACSIN3 | Hs00367625_m1 |
| PI4KB | Hs01090927_m1 |
| PRKD1 | Hs00177037_m1 |
| RAB11A | Hs00900539_m1 |
| RAB3D | Hs00758197_m1 |
| RHOA | Hs00236938_m1 |
| SAR1A | Hs00833068_s1 |
| VAMP1 | Hs00249911_m1 |
| WAS | Hs00166001_m1 |
| ^a^ Applied Biosystems product no. | |
